# Procoxacin bidirectionally inhibits osteoblastic and osteoclastic activity in bone and suppresses bone metastasis of prostate cancer

**DOI:** 10.1186/s13046-023-02610-7

**Published:** 2023-02-09

**Authors:** Depei Kong, Chen Ye, Chenxi Zhang, Xiaochen Sun, Fubo Wang, Rui Chen, Guangan Xiao, Shipeng He, Jianrong Xu, Xiwu Rao, Jianzhong Ai, Xu Gao, Hong Li, Li Su

**Affiliations:** 1 Department of Urology, Institute of Urology, West China Hospital, Sichuan University, 88 South Keyuan Road, Chengdu, 610093 China; 2 Institute of Translational Medicine, Shanghai University, 99 Shangda Road, Shanghai, 200444 China; 3 Department of Urology, Changhai Hospital, Second Military Medical University, Shanghai, 200433 China; 4 Center for Genomic and Personalized Medicine, Guangxi Key Laboratory for Genomic and Personalized Medicine, Guangxi Collaborative Innovation Center for Genomic and Personalized Medicine, Guangxi Medical University, 22 Shuangyong Road, Nanning, 530021 China; 5 Academy of Integrative Medicine, Shanghai University of Traditional Chinese Medicine, 1200 Cailun Road, Shanghai, 201203 China; 6 Department of Pharmacology and Chemical Biology, School of Medicine, Shanghai Jiao Tong University, 280 South Chongqing Road, Shanghai, 200025 China; 7 Department of Medical Oncology and Cancer Institute, Shuguang Hospital, Shanghai University of Traditional Chinese Medicine, Shanghai, 200021 China

**Keywords:** Osteoblast, Osteoclast, Prostate cancer, Metastasis, Drug screening, Procoxacin, Drug target

## Abstract

**Background:**

Bone is the most common site of metastasis of prostate cancer (PCa). PCa invasion leads to a disruption of osteogenic-osteolytic balance and causes abnormal bone formation. The interaction between PCa and bone stromal cells, especially osteoblasts (OB), is considered essential for the disease progression. However, drugs that effectively block the cancer-bone interaction and regulate the osteogenic-osteolytic balance remain undiscovered.

**Methods:**

A reporter gene system was constructed to screen compounds that could inhibit PCa-induced OB activation from 631 compounds. Then, the pharmacological effects of a candidate drug, Procoxacin (Pro), on OBs, osteoclasts (OCs) and cancer-bone interaction were studied in cellular models. Intratibial inoculation, micro-CT and histological analysis were used to explore the effect of Pro on osteogenic and osteolytic metastatic lesions. Bioinformatic analysis and experiments including qPCR, western blotting and ELISA assay were used to identify the effector molecules of Pro in the cancer-bone microenvironment. Virtual screening, molecular docking, surface plasmon resonance assay and RNA knockdown were utilized to identify the drug target of Pro. Experiments including co-IP, western blotting and immunofluorescence were performed to reveal the role of Pro binding to its target. Intracardiac inoculation metastasis model and survival analysis were used to investigate the therapeutic effect of Pro on metastatic cancer.

**Results:**

Luciferase reporter gene consisted of Runx2 binding sequence, OSE2, and *Alp* promotor could sensitively reflect the intensity of PCa-OB interaction. Pro best matched the screening criteria among 631 compounds in drug screening. Further study demonstrated that Pro effectively inhibited the PCa-induced osteoblastic changes without killing OBs or PCa cells and directly killed OCs or suppressed osteoclastic functions at very low concentrations. Mechanism study revealed that Pro broke the feedback loop of TGF-β/C-Raf/MAPK pathway by sandwiching into 14–3-3ζ/C-Raf complex and prevented its disassociation. Pro treatment alleviated both osteogenic and osteolytic lesions in PCa-involved bones and reduced the number of metastases of PCa in vivo.

**Conclusions:**

In summary, our study provides a drug screening strategy based on the cancer-host microenvironment and demonstrates that Pro effectively inhibits both osteoblastic and osteoclastic lesions in PCa-involved bones, which makes it a promising therapeutic agent for PCa bone metastasis.

**Graphical Abstract:**

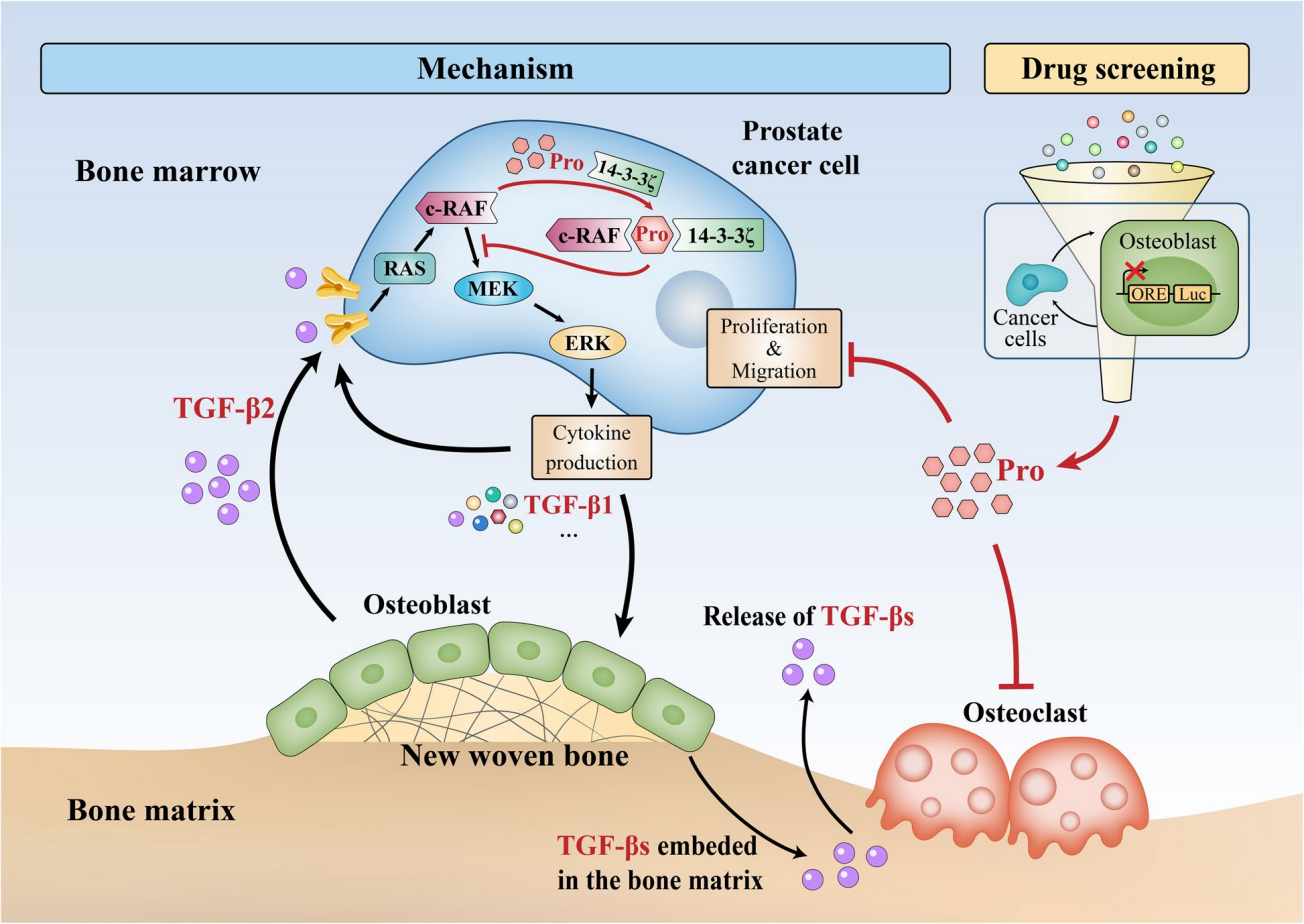

**Supplementary Information:**

The online version contains supplementary material available at 10.1186/s13046-023-02610-7.

## Background

Bone is the most common metastatic site in breast and prostate cancers [1, 2]. The homeostatic balance between osteoclastic bone resorption and osteoblastic bone formation is disrupted in bone metastases, and whether the change is dominated by bone resorption or formation depends on the way cancer cells interact with bone stromal cells. Radiographically, Breast cancer usually causes osteolytic changes in bones while the bone involved by prostate cancer (PCa) usually shows osteogenic changes [3, 4]. Even though, histological evidence shows that the development of bone metastasis involves both osteoclasts (OCs) and osteoblasts (OBs) regardless of cancer types [5]. This implies that drugs that effectively suppress the OC and OB activity in the tumor microenvironment may inhibit the progression of bone metastasis. However, drugs that simultaneously inhibit the osteoclastic and osteoblastic activity in bone metastasis have not been developed so far.

PCa has been the most common malignancy in men, resulting in 1.4 million new cases and 375,000 deaths worldwide in 2020 [6], and approximately 90% of patients who die of PCa develop bone metastases [7]. Conventional cytoreductive therapies such as androgen deprivation and chemotherapy are unable to eradicate PCa cells and the 5-year survival rate of metastatic prostate cancer (mPCa) is around 30% [8]. Patients with bone metastases harbor osteogenic bone lesions and suffer from skeletal-related events (SREs) including hypercalcemia, intractable pain and pathologic fractures. As the interaction between cancer and bone cells has been increasingly recognized as a potential therapeutic target [9, 10], targeting the bone cells in PCa bone metastasis might be a feasible therapeutic approach. However, osteoclast-targeting agents such as bisphosphonates and RANKL antibodies can only delay the occurrence of SREs without suppressing cancer progression [11, 12].

In the case of PCa bone metastasis, OBs recruit cancer cells to the bone surface by expressing various cytokines such as interleukin-6 (IL-6), transforming growth factor beta (TGF-β), insulin-like growth factors (IGFs) and Wnts [13]. In turn, cancer cells promote the maturation of OBs [14, 15], which then activate OCs through the secretion of cytokines such as receptor activators of NF-κB ligand (RANKL). Then, activated OCs cause bone degradation and release growth factors, such as TGF-β, in the bone matrix, which provides positive feedback to cancer cells, forming a “vicious cycle” [2]. In this process, cancer-activated OBs play a predominant role and lead to osteoblastic changes, as histological evidence suggests that PCa cells and OBs are spatially close to each other, and abnormal woven bone is produced by spindle-shaped OBs surrounding the cancer [3, 4].

In this study, we aimed to search for compounds that could block the osteoblastic “vicious cycle” in bone metastasis. Since natural compounds are an important source of anti-tumor drugs and are more accessible, we constructed a drug screening system based on PCa-OB co-culture and screened 631 natural compounds, among which we found Procoxacin (Pro), an ionophore antibiotic also known as salinomycin, to be a promising drug candidate. Pro has been reported to selectively kill breast cancer stem cells [16] and inhibit multiple types of human cancers, including glioblastoma [17], hepatoma [18], ovarian cancers [19] and PCa [20] in vitro. Here, we investigated the effect of Pro on OBs and OCs in the setting of PCa bone metastasis and for the time reported one of the drug targets of Pro and the corresponding drug mechanism.

## Methods

### Cell culture

Human PCa cell lines PC-3, mouse pre-osteoblast cell lines MC3T3-E1 and C3H10T1/2 were purchased from the Shanghai cell bank, Chinese Academy of Sciences. C4-2B cells were kindly provided and authenticated by Dr. Leland Chung (Cedars-Sinai Medical Center, Los Angeles, CA). Human prostate hyperplasia cell line BPH-1 was kindly provided by Dr. Ju Zhang (Bioactive Materials Key Lab of Ministry of Education, Nankai University, China). Mouse bone marrow-derived stroma cells (mBMSCs) were isolated by flushing the tibias and femurs of 6-week-old C57 mice with basal α-MEM. C4-2B, BPH-1 and U937 cells were maintained in RPMI-1640 medium (Gibco) supplemented with 10% fetal bovine serum (FBS, Gibco) and 1% penicillin/streptomycin (P/S, Gibico). PC-3 cells were maintained in F-12 K medium (Gibco) supplemented with 10% FBS and 1% P/S. BMSCs were maintained in α-MEM (Gibco) supplemented with 15% FBS and 1% P/S. Recombinant human TGF-β1 (100–21) was purchased from PeproTech, USA. SB-431542 and PD98059 were purchased from MedChemexpress (MCE, USA) and were both used at a concentration of 10 μM.

### Lentivirus transfection

Lentivirus containing ORE-Luciferase-Puro (vector CV123), EGFP-Hygromycin (vector GV505) and RFP-Puro (vector GV298) genes were purchased from GeneChem (Shanghai, China). Briefly, the promotor region of murine *Alp* was inserted upstream of the luciferase gene and six copies of Runx2 binding site OSE2 oligonucleotides (5’-GATCCGCTGCAATCACCAACCACAGCA-3’) were cloned upstream of *Alp* promotor. ORE-Luciferase-Puro and EGFP-Hygromycin were co-transfected into MC3T3-E1 cells followed by 2-week puromycin (1 µg/ml) and hygromycin (100 µg/ml) selection. RFP-Puro was transfected into C4-2B cells followed by 2-week puromycin (2 µg/ml) selection. Lentivirus (LV16) purchased from GenePharma (Shanghai, China) containing U6-Luciferase-Puro genes (shCtrl) or three shRNA sequences, 5’-GCTGGTTCAGAAGGCCAAACT-3’ (sh140), 5’-GCTAAGAGATATCTGCAATGA-3’ (sh392) and 5’-GGAAATGCAACCAACACATCC-3’ (sh599), were transfected into C4-2B cells followed by 2-week puromycin (2 µg/ml) selection.

### Compounds screening

Compounds library (HY-L021) containing 631 compounds was purchased from MedChemexpress (MCE, USA) and all compounds were pre-dissolved in DMSO (10 mM, 100 μl) by the manufacturer. Before use, 2 µl of pre-dissolved compounds were mixed with 198 µl of DMEM medium in wells of the 96-well plates to prepare compounds at a concentration of 100 µM. Promineralization medium (PM) was made by α-MEM supplemented with 15% FBS, 1% P/S, 100 nM dexamethasone, 10 mM β-glycerophosphate and 100 mM ascorbic acid. For compounds screening, MC3T3-E1 cells were mixed with C4-2B cells at a ratio of 1,2 and 2,000 mixed cells were seeded in each well of 96-well plate. After six hours of incubation in PM, compounds were added into the culture medium at a final concentration of 2 μM for three repeats. Three wells on each plate were set as control by adding DMSO (1/5000). Twenty-four hours later, green and red fluorescence was first detected by the Cytation 5 Cell Imaging Multi-mode Reader (BioTek Instruments, USA) and then luciferin luminescence detection was performed using Luciferase Assay System (Promega, USA).

### Cell viability assay

Cells were seeded in 96-well plates at a density of 2,000 cells per well and treated with DMSO or indicated compounds in three replicates for 48 h. Cell Counting Kit-8 (DOJINDO, Japan) was used to assess the cell viability and samples were measured by Cytation 5 Cell Imaging Multi-mode Reader (450 nm) (BioTek Instruments, USA) according to the manufacturer’s protocol.

### Alizarin Red S staining

At the end of the calcium deposition period (21 days), OBs in the 6-well plate were fixed in 4% paraformaldehyde (PFA) for 15 min at room temperature and washed three times with PBS. Then samples were stained with 2% Alizarin Red S solution (C0138, Beyotime, Chian) at 37℃ for 30 min and washed three times with double-distilled water. Plates were air-dried before imaging under light microscopy. Mineralization intensity was measured using ImageJ software.

### Osteoclast differentiation and bone resorption

BMMs were differentiated in α-MEM supplemented with 10% FBS, 1% P/S, 30 ng/ml M-CSF (R&D Systems) and 50 ng/ml RANKL (R&D Systems). BMMs stimulated with RANKL for 3 days were reseeded onto hydroxyapatite-coated OsteoAssay plates in three replicates and then treated with DMSO or indicated concentrations of Pro for 7 additional days. Cells were removed with sodium hypochlorite, and resorption pits were imaged under light microscopy.

### Animal experiments

All animal studies received prior approval from the Animal Care and Use Committee of Shanghai University. Mice were purchased from Cavens Laboratory Animal Ltd. (Jiangsu Province, China) and fed for an additional week before the experiment. For intratibial injection, 2*10^6^ luciferase-expressing C4-2B (or C4-2B with 14–3-3ζ knockdown) and PC-3 cells in PBS were mixed with Matrigel (354,234, Corning) (1,1, v/v) and injected (total volume 50 μL) into the left or right tibia bone through the tibial plateau of anesthetized Nude mice (male, 6 weeks old). Pro (2 mg/kg) (HY-15597, MCE) or 100μL of PBS was administered intraperitoneally every other day and bioluminescence images were taken each week using IVIS® Lumina III in Vivo Imaging System (PerkinElmer, USA). Mice were euthanized when the diameter of tumor exceeded 1.5 cm or at 90 days after tumor inoculation. Tumor-involved tissue was fixed in 95% ethanol or formaldehyde for following radiographic or pathological examination.

For intracardiac injection, C4-2B (1*10^6^) and PC-3 (1*10^5^) cells stably expressing luciferase were injected into the left ventricle of anesthetized NOD-SCID mice pretreated with Pro (1 mg/kg/day for 3 days) via insulin needle. Ten minutes after injection, bioluminescence of each mouse was detected using IVIS® Lumina III in Vivo Imaging System after an intraperitoneal injection of 150 mg/kg _D_-luciferin (Promega, USA) in 100 μL of PBS. Mice with bioluminescence in lungs or with no bioluminescence were excluded. Three weeks later, bioluminescence of each mouse was photographed. Then, Pro (2 mg/kg) or 100μL of PBS was administered intraperitoneally every other day and survival rate of mice was recorded.

### Micro-CT analysis

Three-dimensional reconstructions of the harvested bone samples (calvarias and tibias) were created from images acquired using the μCT 100 micro-CT scanner (SCANCO, Switzerland). Images were taken at a voltage of 70 kV, current of 200 μA, exposure time of 300 ms and isotropic resolution of 10 mm. Quantitative morphometric analyses of trabecular bone were carried out with a square region of interest (ROI) 0.5 mm below the tibial growth plate.

### Analysis of GEO data

The data of GSE22813 were downloaded from the GEO database, with a total of 8 groups of mRNA transcriptome data, including the control group (n = 5) and the experimental group (n = 3). The annotation platform is GPL8321. The "limma" package was used for differential analysis of the data, and the screening criteria for differential genes were FC > 2 or < 0.5 and adjust P value < 0.05. The differential genes were annotated using the R package "ClusterProfiler" to comprehensively explore the functional relevance of these differential genes. Gene Ontology (GO) and Kyoto Encyclopedia of Genes and Genomes (KEGG) were used to assess related functional categories. GO and KEGG enriched pathways with both p-value and q-value less than 0.05 were considered significant.

### Elisa assay

C4-2B and MC3T3-E1 cells were co-cultured in PM for 6 h. Then, the cells were washed once with PBS and co-cultured in PM without serum. After 18 h, the culture medium was collected and filtered. TGF-β1 (EK981) and TGF-β2 (EK9162) were quantified using an Elisa assay kit (MultiSciences, China) following the manufacturer’s protocol.

### Molecular docking

Molecular docking was performed by the program LeDock_GO. The structure with PDB entry 4IHL and 4DRI were used for the RAF/14–3-3ζ protein complex and FKBP5/mTOR complex, respectively. Crystal waters and co-crystalized ligands in all complex structures were removed, and hydrogen atoms were added to each protein according to the protonation states of the chemical groups at pH 7.0. Metal ions interacting with the crystal ligands were kept during docking. The binding pocket was determined to include any protein atom within at least 5 Å of any atom of the complexed ligand. The 3D structure of Pro was generated by the program CORINA and further minimized with the CHARMM force field.

### Preparation of Procoxacin-conjugated magnetic microbeads

Magnetic microbeads were purchased from Shanghai Yike Biological co., Ltd. To synthesize Procoxacin-conjugated magnetic microbeads, 10 mg amino microbeads, 13.5 mg 1-(3-dimethylaminopropyl)-3-ethylcarbodiimide hydrochloride (EDCl, 0.070 mmol), and 8.6 mg 4-dimethylaminopyridine (DMAP, 0.070 mmol) were added into the solution of 54.1 mg Pro (0.07 mmol) in 20 ml CH_2_Cl_2_. The solution was stirred at room temperature under nitrogen for 24 h and then concentrated under reduced pressure. Microbeads were collected with a magnet, then blocked in 2.5 mg/ml of BSA for 1 h at room temperature and washed with PBS for 3 times to yield the conjugated magnetic beads.

### Surface plasmon resonance (SPR)

SPR was performed on a Biacore T200 instrument (GE Healthcare, USA). 14–3-3ζ (ab87361, Abcam) was captured on an NTA chip. PBS (0.01 M, pH 7.4) served as the buffer solution. 14–3-3ζ solution at 50 μg/mL was injected for 360 s at a flow rate of 10 μL/min. The upstream parallel flow cell was blank (filled with PBS). The binding of different concentrations of the drug (0.195–12.5 μM) was conducted in PBS (0.01 M, pH 7.4) and injected into the flow system for 90 s at a flow rate of 30 μL/min. Dissociation was conducted in the same buffer for 120 s, and there was no regeneration step between injections. Calculation of the binding constant was performed in the BIA evaluation software using the 1,1 Langmuir binding model.

### Antibodies used in Western blot (WB), Immunohistochemistry (IHC) and Immunofluorescence (IF)

The following primary antibodies were used in WB analysis, rat anti-TGF-β2 (771,244, Novus), rabbit anti-TGF-β1 (ab215715, Abcam), 14–3-3ζ (ab51129, Abcam), C-Raf (ab181115, Abcam), phospho-C-Raf (Ser621) (ab157201, Abcam), phospho-C-Raf (Ser259) (ab173539, Abcam), phospho-MEK1/2 (Ser217/221) (#9154, CST), phospho-ERK1/2 (Thr202/Tyr204) (#9101, CST) and GAPDH (#5174, CST) antibodies. The following secondary antibodies were used in WB, anti-rabbit IgG, HRP-linked Antibody (#7074, CST) and anti-rat IgG, HRP-linked Antibody (#7077, CST).

The following antibodies were used in IHC staining, rabbit anti-Cleaved Caspase-3 (#9661, CST), Cyclin D1 (#55,506, CST), TGF-β1 (ab215715, Abcam) and 14–3-3ζ (ab51129, Abcam) antibodies.

The following primary antibodies were used in the IF, rabbit anti-C-Raf (ab181115, Abcam) antibody and mouse anti-14–3-3ζ (abs100410, Absin, China) antibody. The following secondary antibodies were used in IF, Anti-mouse IgG, Alexa Fluor® 594 Conjugate Antibody (#8890, CST) and Anti-rabbit IgG, Alexa Fluor® 488 Conjugate Antibody (#4412, CST).

### WB analysis

In experiments using co-cultured cells, MC3T3-E1 and C4-2B (1,1) were cultured on the lower and upper chambers, respectively, in a Transwell (0.4 μm, #3412, Corning) in PM. Cells were lysed in RIPA buffer (89,901, Thermo Fisher Scientific) containing protease inhibitor (HY-K0010, MCE) and phosphatase inhibitor cocktails (HY-K0021 and HY-K0022, MCE). Then, equal amounts of protein were resolved on 6 to 12% SDS-PAGE and transferred to PVDF membranes (IPVH00010, Millipore). Membranes were incubated with primary antibody overnight at 4 °C. Next, membranes were washed with TBST and incubated with secondary antibodies. Proteins were visualized using ECL detection reagents (1,705,062, Bio-rad.)

### Histomorphometry and IHC analysis

Tumor-bearing tibial bones were excised and fixed in 4% PFA for 3 days, followed by decalcification in 10% EDTA for 2 weeks. Samples were embedded in paraffin and 4 μm sections were cut for hematoxylin and eosin (H&E), alkaline phosphatase (ALP), tartrate-resistant acid phosphatase (TRAP) and IHC staining. Paraformaldehyde fixed paraffin embedded samples were deparaffinized, rehydrated, and subjected to heat-mediated antigen retrieval. The UltraSensitive TM SP (rabbit) IHC Kit (KIT-9706, Fuzhou Maixin Biotech) was used by following the manufacturer’s instructions with minor modification. Briefly, the sections were incubated with 3% H_2_O_2_ for 15 min at room temperature to eliminate the endogenous peroxidase activity. After incubating in normal goat serum for 1 h, sections were treated with primary antibody (diluted as the manufacturer’s instruction) at 4 °C overnight. The sections were then washed 3 times in PBS and treated with biotinylated goat-anti-rabbit IgG secondary antibodies for 30 min followed by incubating with streptavidin-conjugated HRP for 15 min. After washing three times in PBS for 5 min each, specific detection was developed with 3′3-diaminobenzidine (DAB-2031, Fuzhou Maixin Biotech).

### IF analysis

Briefly, C4-2B cells were plated on culture slides (801,010, NEST, China) and treated with DMSO or Pro for 48 h. Then, samples were fixed using 4% PFA solution for 15 min at room temperature and penetrated with 0.8% Triton X-100 (in PBS) for 5 min and blocked by goat serum (C0265, Beyotime) for 30 min. Then, cells were co-incubated with indicated primary antibodies overnight. After being washed in PBS for 3 times, samples were incubated with secondary antibodies (mouse and rabbit) at room temperature for 30 min. Cells were then counterstained with DAPI to visualize nuclear DNA and examined using Nikon imaging system (Ti2-E).

### Real-time PCR

Total RNAs were extracted by RNAiso Plus (9109, Takara, Japan) and reverse transcription was performed using PrimeScript One Step RT reagent Kit (RR037B, Takara, Japan). Real-time PCR was performed using SYBR Green Realtime PCR Master Mix (QPK201, Toyobo, Japan) with ABI PRISM 7300HT (Applied Biosystems, USA) or qTOWER3/G (Analytik Jena, Germany). Genes were measured in two replicates and the relative expression were normalized by GAPDH. The primer sequences were listed in Table S1.

### Statistical analysis

All the experiments in this study were performed with at least two biological repeats except compound screening and animal experiments which were performed once. Statistical analyses were performed using GraphPad PRISM (version 6, Graphpad software Inc.) Data were tested for normality before the use of parametric assessments. Comparisons between the treatment group and control group at specific time points were analyzed by unpaired two-tailed Student’s t test or one-way analysis of variance (ANOVA). Bar graphs display individual data points and report the data as the means ± SEM. *P* < 0.05 was considered statistically significant.

## Result

### Construction of PCa-OB interaction reporter gene system

**Fig. 1:**
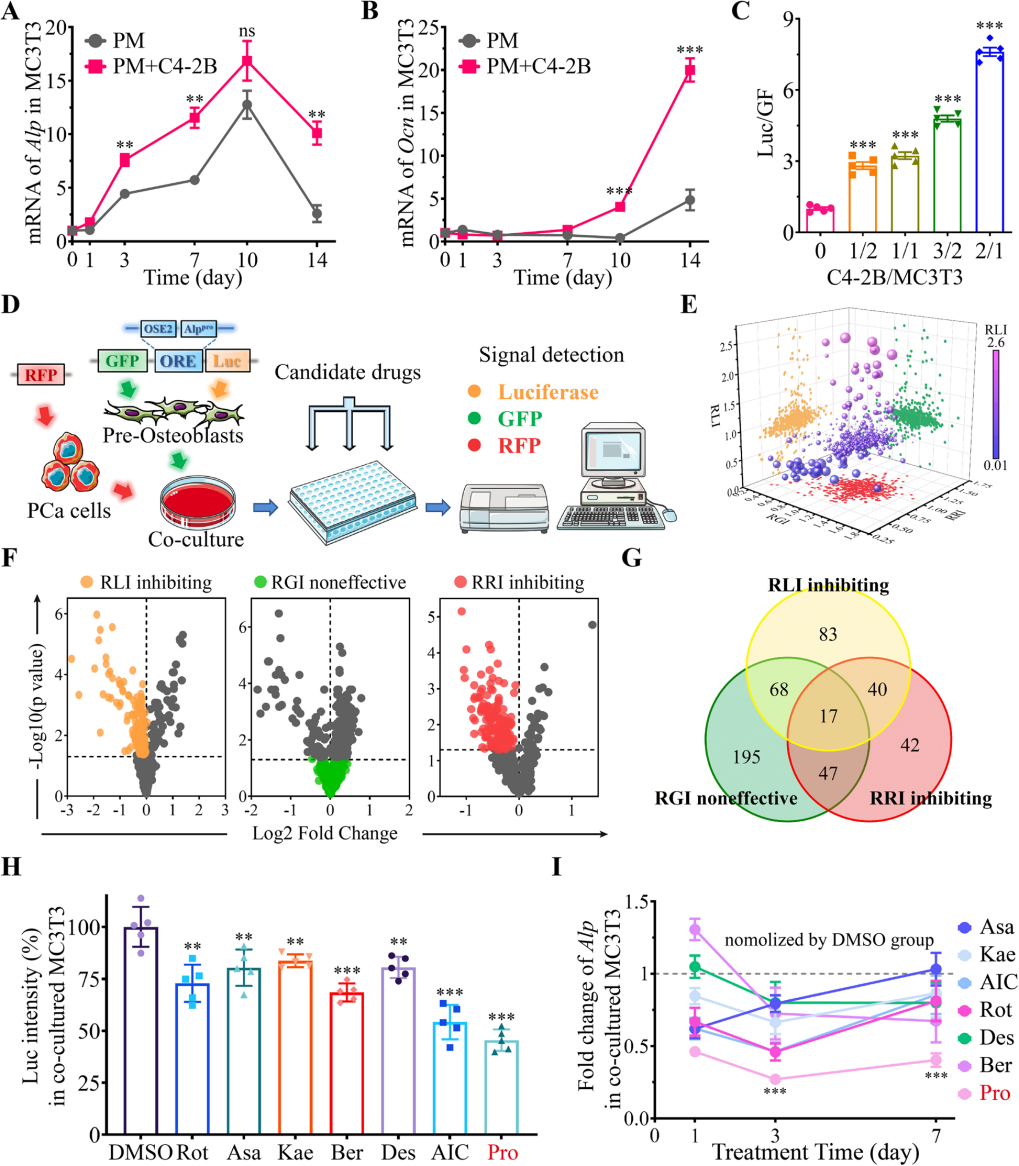
Screening system construction and drug screening. **A** and **B** Relative mRNA expression (normalized by Day 0) of *Alp* (**A**) and *Ocn* (**B**) in MC3T3-E1 cells directly cultured and co-cultured with C4-2B cells in PM (promineralization medium). **C** Normalized luciferase activity (Luc/GF) at 48 h in MC3T3-E1 co-cultured with C4-2B cells at indicated ratios. **D** Schematic of the reporter gene system and screening strategy. **E** Three-dimensional scatter diagram of candidate compounds in accordance with relative green fluorescence intensity (RGI), relative red fluorescence intensity (RRI) and relative luciferin luminescence intensity (RLI) for each compound. The larger dot indicates the smaller *P*-value. **F** Volcano plots show compounds that have suppressive effects on RLI (orange dots) and RRI (red dots), as well as compounds that have no effect on RGI (green dots). **G** Overlap between the RLI inhibiting group, RGI non-effecting group and RRI inhibiting group. **H** Relative luciferin luminescence intensity in ME3T3-ORE co-cultured with C4-2B cells treated with indicated compounds (2 μM, 48 h). **I** Fold change of *Alp* mRNA at different time points compared with Day 0 in ME3T3-E1 co-cultured with C4-2B cells and treated with DMSO or indicated compounds (2 μM). *P < 0.05, **P < 0.01, ***P < 0.001, ns, no significance

Given the fact that bone metastasis of PCa is osteogenic and OC suppression cannot suppress cancer progression, we decided to take the PCa-OB interaction as a target for drug screening. We first checked the expression of several important osteoblastic genes in OBs cultured with or without PCa cells. Transcriptional levels of *Runx2*, *Ocn*, *Col1a1*, *Opn* and *Alp* were examined in osteoblastic lineage cell line MC3T3-E1 cultured in basal medium or promineralization medium (PM) with or without a bone-derived mPCa cell line, C4-2B. In PM-cultured MC3T3-E1 cells, the mRNA level of *Alp* was significantly increased and peaked at day 10 compared with day 0. In addition, the elevation of *Alp* became more pronounced after the addition of C4-2B cells (Fig. 1A). By contrast, the mRNA level of *Ocn* remained unchanged until day 7 and began to increase after day 10 (Fig. 1B). No significant elevation was seen in the expression of *Runx2*, *Opn* and *Col1a1* in co-cultured MC3T3-E1 cells within 7 days, as shown in Fig. S1A, S1B and S1C, respectively. Thus, we used *Alp* as a reporter gene and introduced murine *Alp* promotor sequence into the promotor region of the luciferase gene.

In addition, though the mRNA level of *Runx2* was not increased, expression of genes under the transcriptional regulation of Runx2, such as Ocn and Opn, were upregulated. Thus, six copies of Runx2 binding sequence (osteoblast-specific cis-acting element, OSE2) were cloned upstream according to the protocols from Patricia [21] and Yang [22]. The combination of OSE2 sequence and *Alp* promoter is termed the Osteogenesis-response element (ORE) in our work and the reporter gene was stably transfected into MC3T3-E1 cells to establish the MC3T3-ORE cells. Given the number of MC3T3-ORE cells also influence the total luciferase activity, green fluorescence protein (GFP) was transfected into MC3T3-ORE cells as the internal reference.

Then, we verified the reliability of our reporter gene system. MC3T3-ORE (1,000 cells) were co-cultured with different ratios of C4-2B cells in PM. Luciferin luminescence (Luc) and green fluorescence (GF) were detected after 48 h of co-culture. As shown in Fig. 1C, normalized Luc intensity (Luc/GF) increased with the higher number of C4-2B cells.

### High-throughput screening for compounds that inhibit PCa-induced OB activation

Before we screened a library containing 631 natural compounds, in order to assess the effect of each compound on cancer cell viability, red fluorescence protein (RFP) was stably transfected into C4-2B cells (C4-2B-RFP). As shown in the compounds screening schematic (Fig. 1D), the green fluorescence intensity represents the number of MC3T3-ORE cells; the red fluorescence intensity represents the number of C4-2B-RFP cells and the luciferin luminescence intensity represents the osteoblastic activity in MC3T3-ORE cells. MC3T3-ORE and C4-2B-RFP cells were mixed in a ratio of 1:2 and co-cultured in PM. Six hours later, compounds were added into culture medium at a final concentration of 2 μM. After 24 h, green fluorescence, red fluorescence and luminescence signal were collected from each well, and relative green fluorescence intensity (RGI), relative red fluorescence intensity (RRI), and relative fluorescein luminescence intensity (RLI) were calculated by normalizing to control wells (Table S2). The effects of each compound on RGI, RRI and RLI were shown in a three-dimensional coordinate system and projected into a two-dimensional coordinate system (Fig. 1E). We highlighted compounds that downregulated RLI and RRI, and had no effect on RGI in orange, red and green, respectively (Fig. 1F). By intersecting these three groups, 17 compounds (details shown in Table 1) were found, as shown in Venn graph (Fig. 1G). These 17 compounds were believed to be effective in inhibiting PCa-induced OB activation and PCa viability without cytotoxicity on OBs. Notably, AICAR (phosphate) (AIC) and Procoxacin (Pro) could reduce RLI by about 50%. In addition, Ursonic acid and Ginsenoside Rg2 (Gin) reduced the RRI by 45% exhibiting a strong antitumor effect.

**Table 1:** Information of the compounds that meet the screening criteria

Drug Name	RLI	RLI SD	RGI	RGI SD	RRI	RRI SD	M.Wt	Formula	Known Targets
AICAR (phosphate)	0.48	0.02	0.96	0.06	0.84	0.04	356.23	${\mathrm{C}}_{9}{\mathrm{H}}_{17}{\mathrm{N}}_{4}{\mathrm{O}}_{9}\mathrm{P}$	Unknown
Procoxacin	0.54	0.02	0.98	0.07	0.82	0.02	751.00	${\mathrm{C}}_{42}{\mathrm{H}}_{70}{\mathrm{O}}_{11}$	Unknown
Asaraldehyde	0.72	0.03	1.08	0.03	0.69	0.04	196.20	${\mathrm{C}}_{10}{\mathrm{H}}_{12}{\mathrm{O}}_{4}$	Unknown
Bergapten	0.72	0.04	1.09	0.05	0.71	0.02	216.19	${\mathrm{C}}_{12}{\mathrm{H}}_{8}{\mathrm{O}}_{4}$	Cytochrome P450 [23]
Kaempferol	0.73	0.05	1.07	0.07	0.81	0.09	286.24	${\mathrm{C}}_{15}{\mathrm{H}}_{10}{\mathrm{O}}_{6}$	Estrogen Receptor [24]
Rotundine	0.76	0.05	1.02	0.08	0.69	0.15	355.43	${\mathrm{C}}_{21}{\mathrm{H}}_{25}{\mathrm{NO}}_{4}$	Dopamine/5-HT Receptor [25]
Desaminotyrosine	0.77	0.03	0.99	0.02	0.69	0.09	166.18	${\mathrm{C}}_{9}{\mathrm{H}}_{10}{\mathrm{O}}_{3}$	Unknown
Citric acid	0.82	0.01	0.95	0.09	0.60	0.06	281.98	${\mathrm{C}}_{6}{\mathrm{H}}_{13}{\mathrm{Li}}_{3}{\mathrm{O}}_{11}$	Unknown
Tetrahydrobiopterin	0.83	0.05	0.93	0.04	0.67	0.07	241.25	${\mathrm{C}}_{9}{\mathrm{H}}_{15}{\mathrm{N}}_{5}{\mathrm{O}}_{3}$	Unknown
Cryptotanshinone	0.84	0.06	0.91	0.09	0.71	0.15	296.36	${\mathrm{C}}_{19}{\mathrm{H}}_{20}{\mathrm{O}}_{3}$	Unknown
Ursonic acid	0.84	0.01	1.13	0.01	0.55	0.06	454.68	${\mathrm{C}}_{30}{\mathrm{H}}_{46}{\mathrm{O}}_{3}$	Unknown
(-)-Sparteine	0.84	0.01	1.07	0.09	0.61	0.10	234.39	${\mathrm{C}}_{15}{\mathrm{H}}_{26}{\mathrm{N}}_{2}$	Unknown
Loteprednol Etabonate	0.86	0.02	1.01	0.02	0.87	0.04	466.95	${\mathrm{C}}_{24}{\mathrm{H}}_{31}{\mathrm{CIO}}_{7}$	Glucocorticoid Receptor [26]
[6] -Gingerol	0.86	0.04	1.13	0.10	0.66	0.05	294.39	${\mathrm{C}}_{17}{\mathrm{H}}_{26}{\mathrm{O}}_{4}$	Unknown
Deoxycholic acid	0.89	0.03	1.11	0.06	0.68	0.10	392.57	${\mathrm{C}}_{24}{\mathrm{H}}_{40}{\mathrm{O}}_{4}$	Unknown
Ginsenoside Rg2	0.90	0.03	1.10	0.09	0.55	0.08	785.01	${\mathrm{C}}_{42}{\mathrm{H}}_{72}{\mathrm{O}}_{13}$	TLR4 [27]
( +)-Borneol	0.92	0.03	1.07	0.12	0.61	0.08	154.25	${\mathrm{C}}_{10}{\mathrm{H}}_{18}\mathrm{O}$	GABA Receptor [28]

*M.Wt* Molecular Weight

### Evaluating procoxacin as a candidate drug for osteogenic bone metastasis

Of the 17 compounds, 7 compounds with RLI inhibition rate higher than 20% were selected for further validation. As shown in Fig. 1H, Pro (2 μM, 48 h) showed the best suppressive effect on the expression of the luciferase reporter gene. Consistently, expression of *Alp* in MC3T3-E1 cells co-cultured with C4-2B was the lowest in Pro treated group (Fig. 1I). CCK-8 assay (48 h) confirmed that these compounds had little effect on the viability of osteoblastic lineage cell lines MC3T3-E1 (Fig. S1D) and C3H10T1/2 (Fig. S1E), while they suppressed the viability of bone-derived PCa cell lines, C4-2B (Fig. S1F) and PC-3 (Fig. S1G). Taken together, we identified Pro as the best suppressor for PCa-induced OB activation without affecting the viability of OBs, which implied that Pro might inhibit osteogenic bone metastasis with minimal side effects on normal stem cells in the bone marrow.

### Effect of Procoxacin on OB activity

**Fig. 2:**
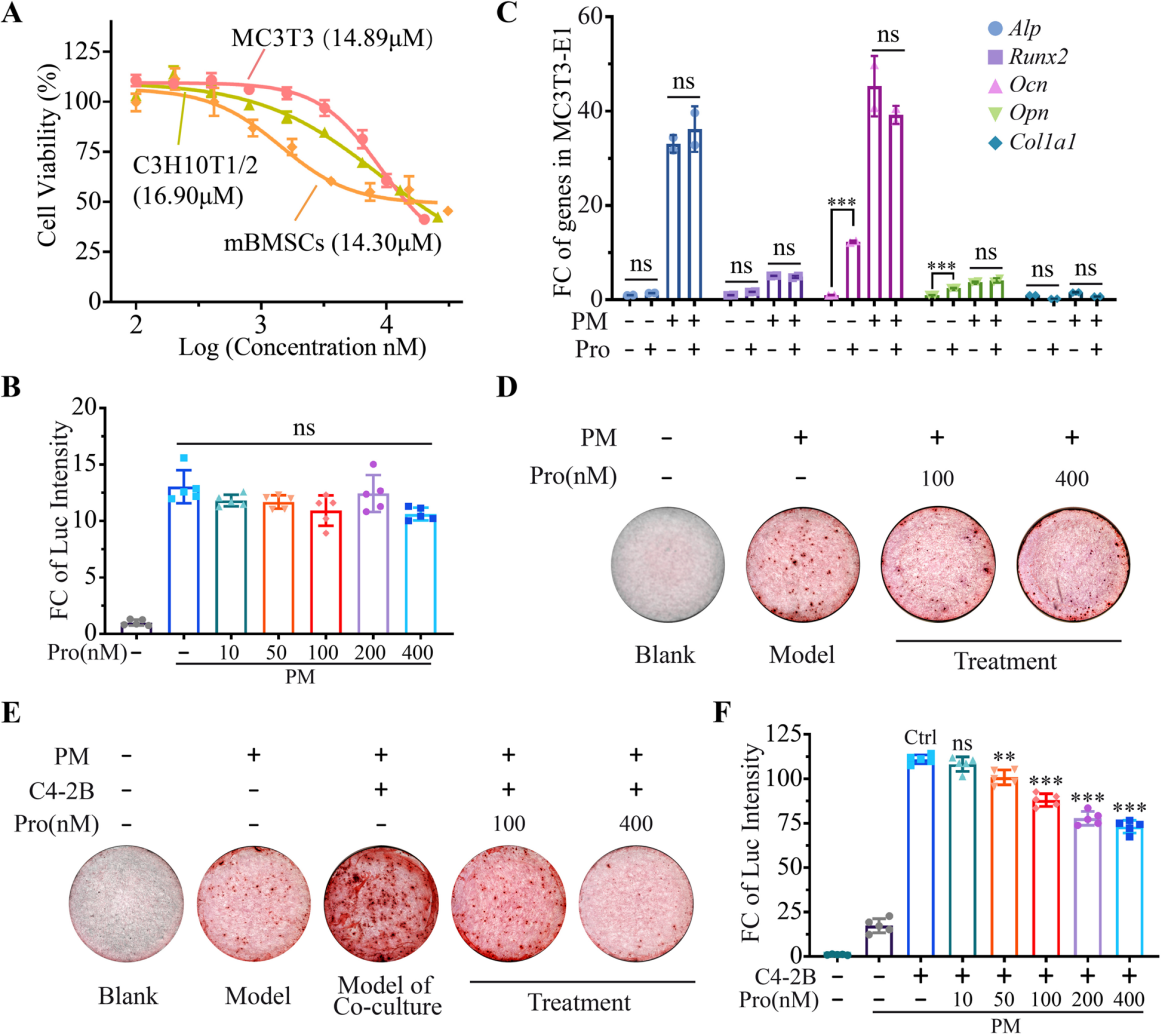
Pro specifically inhibits cancer-induced OB activation and osteogenesis. **A** Cell viability of MC3T3-E1, C3H10T1/2 and mBMSCs with 48-h IC_50_ values indicated. **B** Luciferin luminescence intensity (RLI) in MC3T3-ORE cells treated with Pro at indicated concentrations. **C** Effect of Pro (400 nM) on mRNA level of osteoblastic marker genes in MC3T3-E1 cells cultured in basal medium or PM. **D** and **E** Alizarin Red S staining in directly cultured (**D**) or co-cultured (**E**) MC3T3-E1 cells treated with or without Pro for 21 days. (F) RLI in co-cultured MC3T3-ORE cells treated with Pro at indicated concentrations. **P < 0.01, ***P < 0.001, ns, no significance

The above results showed that Pro at a concentration of 2 μM was effective in killing PCa cells. Thus, the inhibitory effect of Pro on PCa-OB interaction may be a result of PCa cell reduction. However, according to the data from compound screening, only 57 out of 146 PCa-suppressing compounds could inhibit PCa-induced OB activation (Fig. S2A). This suggests that there are other mechanisms behind the inhibitory effect of Pro on OB activation. To avoid PCa cell reduction in the co-culture system, we first tested the cytotoxicity of Pro in C4-2B cells and found that Pro at concentrations lower than 400 nM would not inhibit the viability of C4-2B cells (Fig. S2B). We then investigated the safe concentration of Pro in OBs. Results showed that Pro had a wide range of safe concentration in osteoblastic lineage cells, such as MC3T3-E1, C3H10T1/2 and primary mouse bone marrow mesenchymal stem cells (mBMSCs), as shown in Fig. 2A. Thus, Pro at a maximum concentration of 400 nM was used in the following study. Next, we demonstrated that Pro had little effect on the activation of MC3T3-ORE cells directly cultured in PM (Fig. 2B). At the transcription level, Pro (400 nM) had no significant suppressive effect on osteogenic genes such as *Alp*, *Runx2*, *Ocn* and *Opn*, but instead promoted the expression of *Ocn* and *Opn* in MC3T3-E1 cells cultured without PM (Fig. 2C). Consistently, Alizarin Red S staining showed that Pro (100 and 400 nM) had nearly no effect on the calcium deposition ability of MC3T3-E1 directly cultured in PM for 21 days, as shown in Fig. 2D and quantified in Fig. S3A.

Given that Pro had no obvious effect on directly cultured OB, we proposed that the effect of Pro on osteoblastic activity is cancer-dependent. As shown in Fig. 2E and quantified in Fig. S3B, Pro at concentrations as low as 100 nM could suppress calcium deposition of MC3T3-E1 cultured in PM with C4-2B cells. Similarly, Pro at concentrations higher than 50 nM significantly suppressed the activation of MC3T3-ORE cells co-cultured with C4-2B in PM (Fig. 2F).

### Effect of Procoxacin on OC activity

**Fig. 3:**
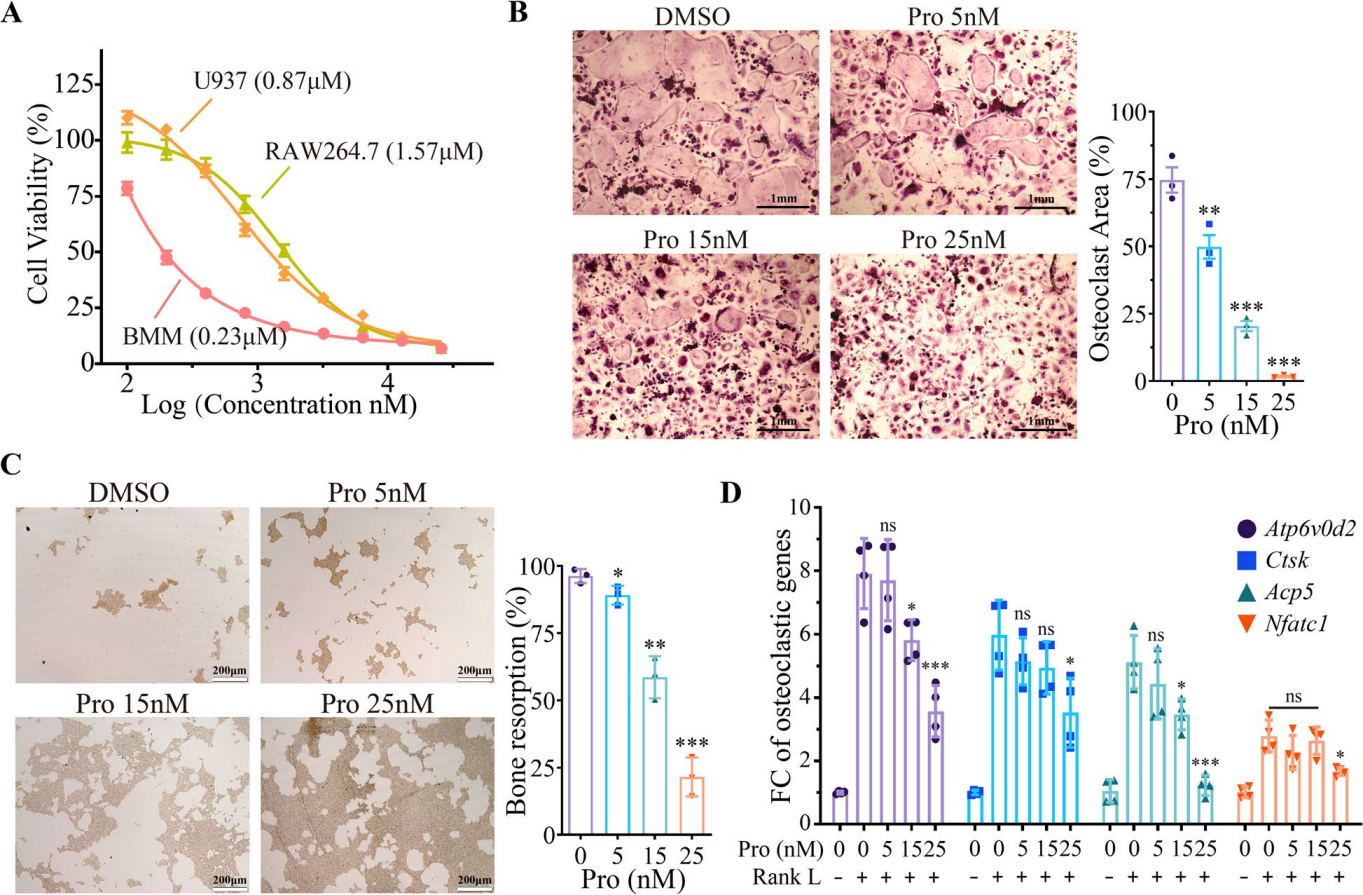
Pro suppresses viability and maturation of osteoclasts. **A** Cell viability of U937, RAW264.7 cells and primary bone marrow macrophages (BMMs) treated with gradient concentrations of Pro. IC_50_ values of Pro in each cell type are indicated on the graph. **B** Representative TRAP staining images of BMMs-derived osteoclasts stimulated by RANKL for 14 days treated with DMSO or Pro at indicated concentrations. The area of mature OCs was quantification on the right. **C** Representative images of bone resorption by BMMs-derived osteoclasts treated with DMSO or Pro at indicated concentrations. The area of absorbed bone was quantification on the right. **D** Fold change of mRNA level of osteoclast marker genes in BMMs in indicated conditions. *P < 0.05, **P < 0.01, ***P < 0.001, ns, no significance

We next examined whether Pro had any effects on OCs. CCK-8 assay showed that pre-osteoclast cells including U937, RAW264.7 and primary bone marrow macrophages (BMMs) were significantly suppressed by Pro with 48-h IC_50_ values of 0.87 μM, 1.57 μM and 0.23 μM, respectively (Fig. 3A). OC differentiation assay by TRAP staining showed that the number and area of TRAP positive multinucleated OCs were significantly reduced by Pro at concentrations as low as 5 nM (Fig. 3B). Consistently, the bone resorption capacity of OCs on bone-mimicking hydroxyapatite coated substrate plates was reduced by Pro in a dose-dependent manner, as shown in Fig. 3C. Consistently, Pro effectively suppressed the expression of several important genes implicated in osteoclastic maturation and function, but had little effect on the expression of *Nfatc1*, a master regulatory transcription factor in OC (Fig. 3D). These results indicate that Pro is a potent inhibitor of viability and function of OCs.

### Therapeutic effect of Procoxacin on both osteogenic and osteolytic metastases of PCa in vivo

**Fig. 4:**
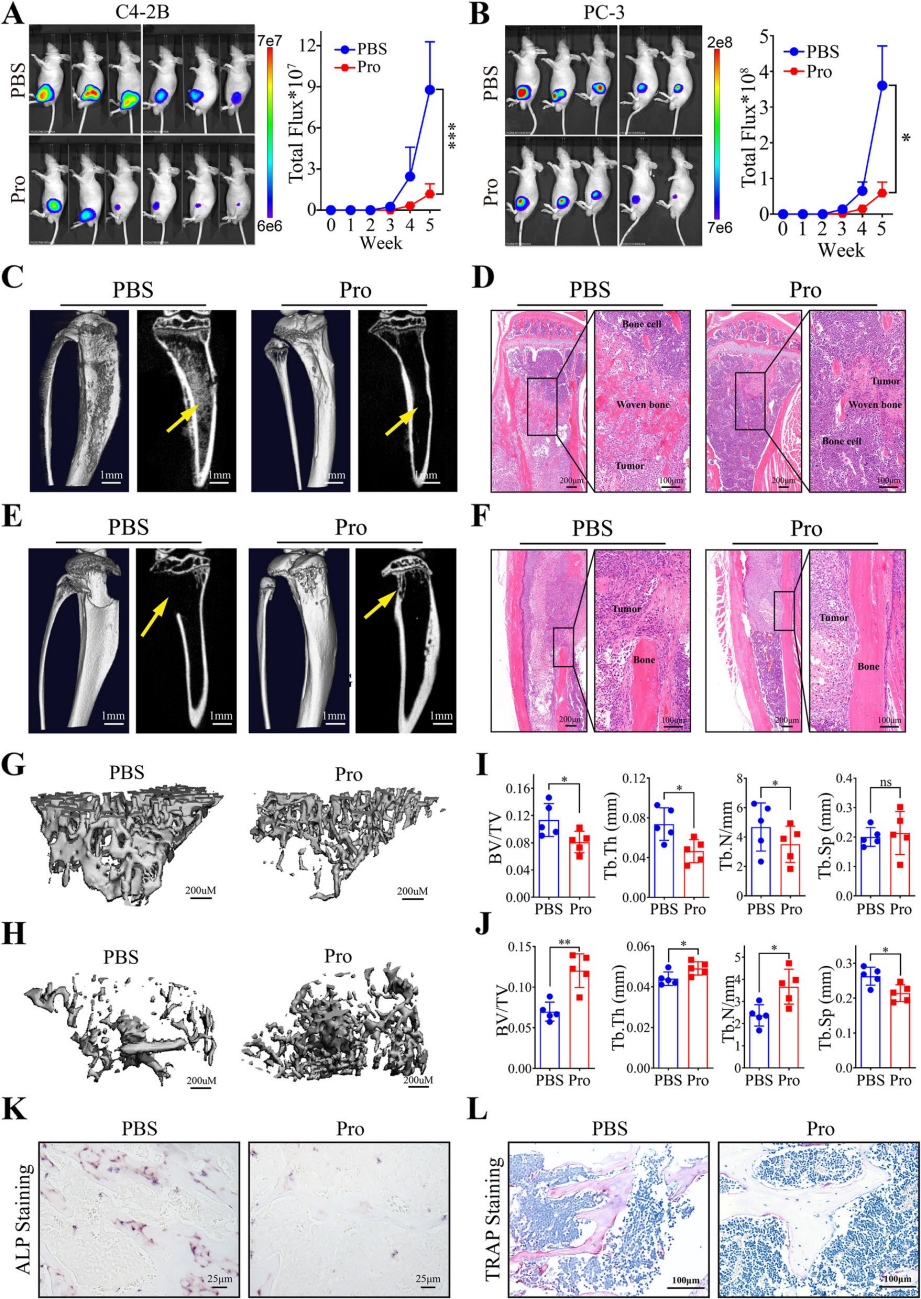
Pro inhibits bone-metastatic PCa cells in vivo. **A** and **B** Representative images for Nude mice injected with C4-2B (**A**) and PC-3 (**B**) in tibias and treated with PBS or Pro. Bioluminescence was quantified on the right. **C** and **D** Representative 3D micro-CT reconstructions (**C**) and H&E staining (**D**) of C4-2B-involved tibias in PBS or Pro treated group. Yellow arrows indicate aberrant woven bone formation in the bone marrow. Scale bars are indicated in the lower right corner. **E** and **F** Representative 3D micro-CT reconstructions (**E**) and H&E staining (**F**) of PC-3-involved tibias in PBS or Pro treated group. Yellow arrows indicate the bone destruction caused by cancer cells. **G** and **H** Representative images of separated trabecular bones in tibias involved by C4-2B (**G**) and PC-3 (**H**) and treated with PBS or Pro. **I** and **J** Quantitative bone morphometric parameters of bone volume to total tissue volume (BV/TV), trabecular bone thickness (Tb.Th), trabecular number (Tb.N., mm^−1^) and trabecular spacing (Tb.Sp., mm) measured in bones involved by C4-2B (**I**) and PC-3 (**J**). **K** ALP staining in bone sections from C4-2B-involved tibias treated with PBS or Pro. **L** TRAP staining in bone sections from PC-3-involved tibias treated with PBS or Pro. *P < 0.05, **P < 0.01, *** *P* < 0.001, ns, no significance

In order to investigate whether Pro could treat PCa bone metastasis in vivo, we established intratibial inoculation mouse models using two bone-derived PCa cell lines, C4-2B and PC-3. As shown in Fig. 4A and B, Pro significantly reduced the tumor burden of Nude mice inoculated with C4-2B or PC-3 cells. Micro-CT and histological analysis showed that C4-2B cells caused aberrant bone formation in the bone marrow, which was significantly suppressed by Pro (Fig. 4C and D). In contrast, PC-3 cells cause significant bone destruction, which could also be ameliorated by Pro (Fig. 4E and F). To distinguish cancer cells and bone stromal cells, CK8/18 was stained and shown in Fig. S4A. We also observed that cancer cells broke the bone cortex and invaded extraskeletal tissues in some of the mice in the control groups (Fig. S4B). Representative images of trabecular bone in C4-2B and PC-3 involved bones with or without Pro treatment were shown in Fig. 4G and H, respectively. Moreover, Pro significantly decreased the percent bone volume (Bone volume/Total volume, BV/TV), trabecular thickness (Tb.Th) and trabecular number (Tb.N), while little difference was observed in trabecular spacing (Tb.Sp) of C4-2B-invovled bones (Fig. 4I). In tibias involved by PC-3 cells, all parameters were significantly improved by Pro treatment (Fig. 4J). Alp staining of C4-2B involved bones and TRAP staining of PC-3 involved bones showed that the activity of both bone formation and bone resorption was suppressed by Pro in vivo (Fig. 4K and L).

### Inhibition of PCa-induced OB activation by Procoxacin via decreasing cancer-derived TGF-β1

**Fig. 5:**
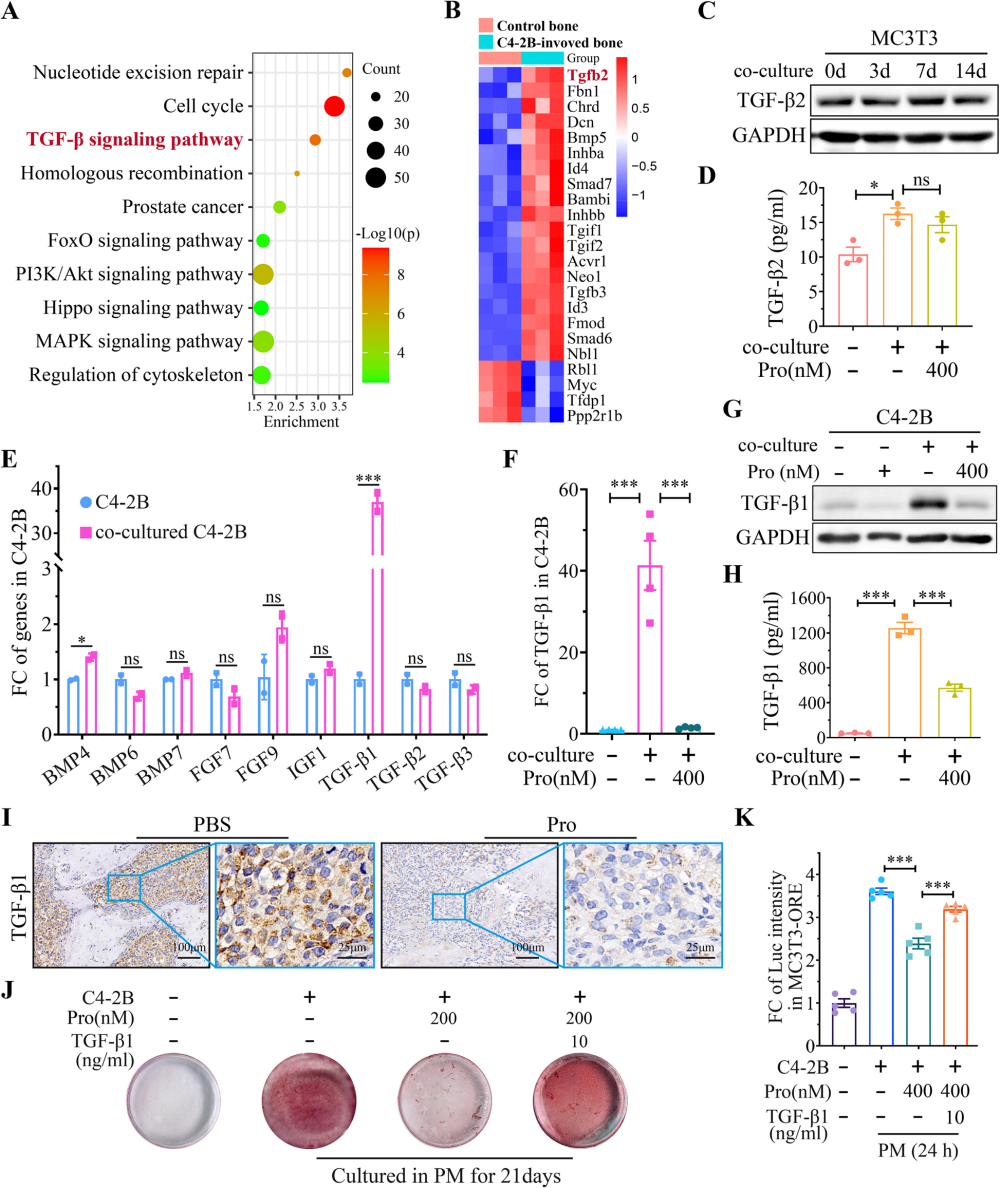
Cancer-derived TGF-β1 is an effector target of Pro. **A** Pathways enriched in C4-2B-involved bone stromal cells. **B** Heat map of differential genes in TGF-β pathway. **C** Western blot of TGF-β2 in MC3T3-E1 cells co-cultured with C4-2B at indicated time points. **D** Concentration of TGF-β2 in medium from MC3T3-E1 at indicated condition at 24 h. **E** Fold change of mRNA level of indicated genes in directly cultured or co-cultured C4-2B cells (48 h). **F** Fold change of mRNA level of TGF-β1 in co-cultured C4-2B treated with Pro (400 nM, 48 h). **G** Western blot of TGF-β1 in co-cultured C4-2B cells treated with Pro (400 nM, 24 h). **H** Elisa assay of TGF-β1 in conditioned medium from directly cultured or co-cultured C4-2B with or without Pro (400 nM, 24 h). **I** IHC staining of TGF-β1 in C4-2B-invovled tibias in PBS or Pro treated group. **J** Alizarin Red S staining in MC3TC3-E1 under indicated conditions at 21 days. **K** Fold change of luciferin luminescence intensity (RLI) in MC3T3-ORE cells treated with Pro (400 nM) and supplemented with TGF-β1 (10 ng/ml) at 24 h. *P < 0.05, **P < 0.01, *** *P* < 0.001, ns, no significance

The present results show that Pro directly inhibits OCs, which may well explain the inhibitory effect of Pro on osteolytic bone metastasis in vivo. However, it is still unclear how Pro blocks the PCa-induced OB activation. To answer this question, we analyzed a data set in Gene Expression Omnibus (GEO) database numbered GSE22813, in which C4-2B cells were injected into mouse tibias and the transcriptome of stroma cells in tumor-involved bones was acquired. By comparing the C4-2B-involved bones with sham-operated bones and intact bones, we found that the TGF-β signaling pathway (Fig. 5A) and transcription of *Tgfb2* (Fig. 5B) in bone stromal cells were significantly activated by C4-2B cells. WB validated that C4-2B induced the expression of TGF-β2 in MC3T3-E1 cells at day 7 (Fig. 5C). However, ELISA assay showed that Pro treatment did not significantly reduce the concentration of TGF-β2, which was around 15 ng/ml, in the co-culture supernatant (Fig. 5D). Also, WB showed a similar result in TGF-β2 expression in MC3T3-E1 cells (Fig. S5).

Given the weak effect of Pro on OBs, we switched our research target to PCa cells. It has already been demonstrated by antibody microarray that PCa cells induce ectopic bone formation by releasing a variety of cytokines, including bone morphogenetic proteins (BMPs), fibroblast growth factors (FGFs), insulin-like growth factors (IGFs), growth differentiation factors (GDFs) and TGF-β [29, 30]. In our study, we examined the expression of BMP, FGF, IGF, and TGF family genes in C4-2B cells co-cultured with MC3T3-E1 and found that *TGF-β1* was elevated dozens of times after 48 h of co-culture (Fig. 5E). We then examined whether Pro could inhibit TGF-β1 expression in co-cultured C4-2B cells using qPCR, WB, and ELISA, and the results showed that Pro dramatically decreased TGF-β1 expression and secretion (Fig. 5F, G and H). Similar result was observed in IHC staining which showed a significant decrease of TGF-β1 in cancer cells in tibias treated with Pro (Fig. 5I).

Since the amount of TGF-β1 produced by C4-2B cells was hundreds of times higher than TGF-β2 produced by OBs, as shown by the results of the ELISA assay, we speculated that TGF-β1 was the key effector target of Pro in suppressing C4-2B-induced MC3T3 activation. Thus, we added Pro together with 10 ng/ml of TGF-β1 to the co-culture system and found that supplementation of TGF-β1 restored the calcium deposition ability of MC3T3-E1 cells (Fig. 5J). Consistently, supplementation of TGF-β1 (10 ng/ml, 24 h) restored the expression of luciferase reporter gene in co-cultured MC3T3-ORE cells in the presence of Pro (Fig. 5K). Taking together, these results indicate that Pro may target PCa-derived TGF-β1 in the bone microenvironment and thus suppress the OB activation.

### In silicon screening of potential drug targets of Procoxacin

Here, though we have found the effector target of Pro to be TGF-β1, it remains unclear in what way Pro regulates TGF-β1 expression in the PCa-OB microenvironment. To date, no molecular targets of Pro have been identified. To uncover the potential drug targets, Pro was queried against the crystal ligands in the Protein Data Bank (PDB). By a Dice similarity threshold of 0.35 with the Morgan fingerprint and 0.8 with a topological pharmacophore fingerprint, five distinct ligands together with their associated proteins were obtained, namely RAF/14–3-3ζ protein complex, FKBP/mTOR complex, protein phosphatase PP1, nuclear receptor RORγ and Actin. To identify the putative protein targets at the atomic level, molecular docking was carried out against these five plausible proteins. The result showed that Pro had weak to modest binding affinity against Actin, nuclear receptor RORγ, and protein phosphatases. Of note, many natural compounds modulate the activity of phosphatases [31]. In contrast, Pro was predicted to be sandwiched into the RAF/14–3-3ζ complex and FKBP/mTOR complex with ΔG values of -9.4 kcal/mol and -9.1 kcal/mol, respectively, showing relatively high binding affinities (Table 2).

**Table 2:** Predicted targets of Procoxacin by virtual screening

Target	Ligand	Similarity^a^	PBD ID	ΔG^b^
RAF/14-3-3ζ complex		0.87/0.41	4IHL	-9.4
FKBP/mTOR complex		0.83/0.35	4DRI 4DRJ 4DRH	-9.1
Protein phosphatase PP1		0.94/0.43	1JK7 1U32	-7.5
Nuclear receptor RORγ		0.86/0.35	3B0W	-6.6
Actin		0.81/0.37	2ASP	-5.7

^a^Dice similarity coefficient with the topological pharmacophore fingerprint and Morgan fingerprint, respectively

^b^Predicted by molecular docking of Pro with targets in kcal/mol

### Identification of 14–3-3ζ as one of drug targets of Procoxacin

**Fig. 6:**
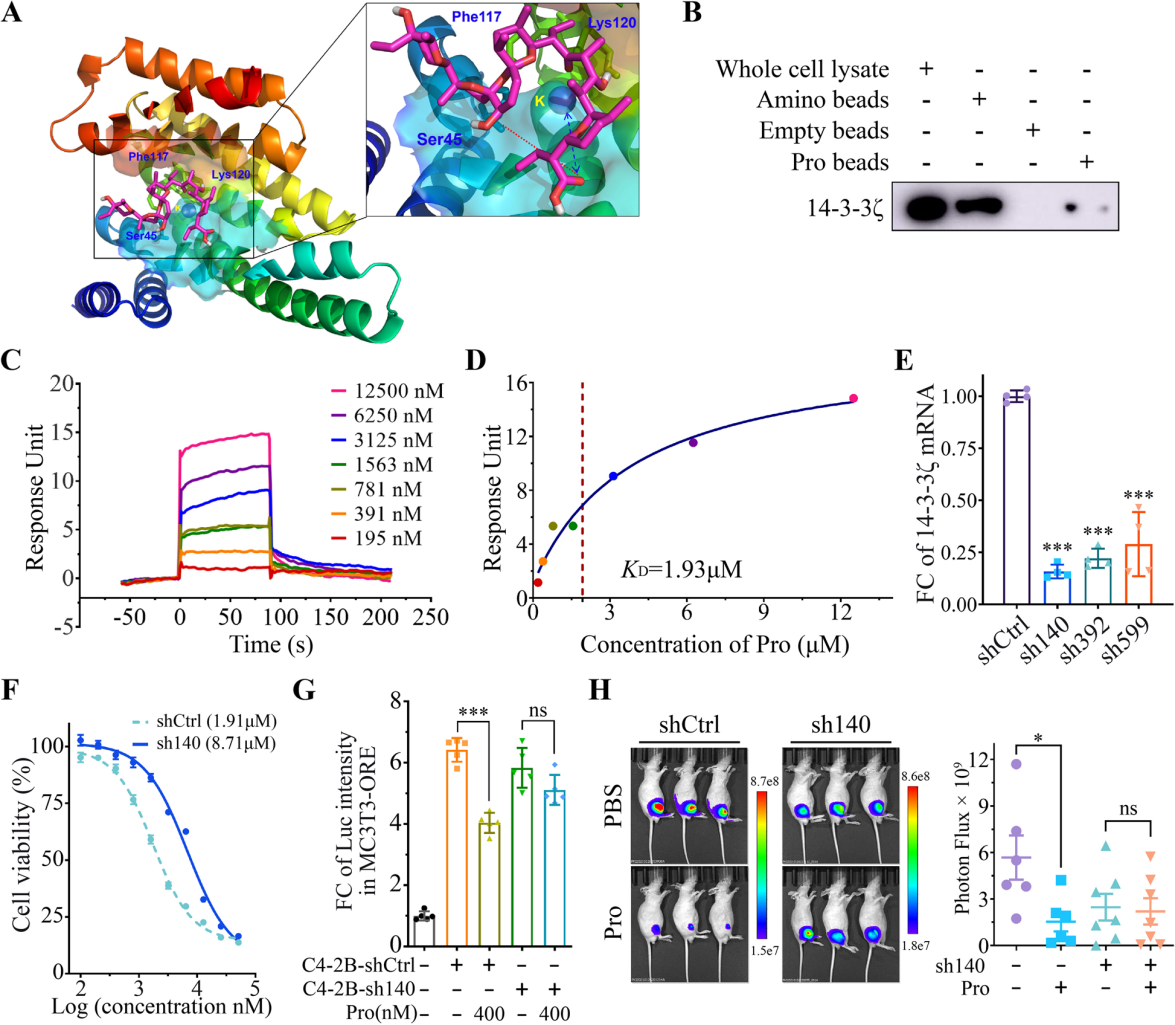
14–3-3ζ is identified as a drug target of Pro. **A** Predicted 3D structure of Pro binding to 14–3-3ζ. **B** Western blot of 14–3-3ζ in primary cell lysate and samples separated by amino magnetic microbeads (A Beads, positive control), empty magnetic microbeads (E beads, negative control) and Pro-conjugated magnetic microbeads (P Beads). **C** Overlay plot of sensorgrams of the Pro/14–3-3ζ binding in SPR. **D** Fit curve of dose-dependent saturation binding of Pro to 14–3-3ζ with dissociation constant (*K*_D_) indicated. **E** Knockdown of 14–3-3ζ in C4-2B with three shRNAs. **F** Viability of shCtrl- or sh140-transfected C4-2B cells treated with Pro. The IC_50_ values were given in parentheses. **G** Relative luciferin luminescence intensity (RLI) in MC3T3-ORE cells under indicated conditions. **H** Representative bioluminescence images of mice bearing C4-2B-shCtrl or C4-2B-sh140 cells treated with PBS or Pro (n=6). Bioluminescence was quantified on the right. *P < 0.05, ***P < 0.001, ns, no significance

It is predicted that the carboxylic group of Pro interacts with the potassium ion at the RAF/14–3-3ζ interface, and forms a salt bridge with Lys120 and an H-bond with Ser45 of 14–3-3ζ. The adjacent ethyl motif inserts deeply into a hydrophobic cleft between Lys120 and Phe117 in 14–3-3ζ (Fig. 6A). To confirm the binding of Pro to 14–3-3ζ, we performed a magnetic beads pull-down assay in C4-2B cells. As shown in Fig. 6B, a weak 14–3-3ζ band in the Pro-conjugated magnetic beads (P Beads) group was observed, while no band was seen in the empty magnetic beads (E Bead, negative control). Furthermore, surface plasmon resonance (SPR) assay provided the direct evidence of Pro binding to 14–3-3ζ (Fig. 6C) with a dissociation constant (KD) of 1.93 μM (Fig. 6D).

Besides, the predicted binding of Pro to the FKBP/mTOR complex is shown in Fig. S6. Briefly, its carboxylic end is anchored on FKBP5 by inserting into a hydrophobic cavity, mainly delineated by aromatic side chains. The spirocycles hold the FRB domain of mTOR onto the surface of FKBP5.

Next, we tested if 14–3-3ζ could regulate the drug effect of Pro by 14–3-3ζ knockdown with shRNAs. Three shRNAs with the best knockdown efficiency of sh140 were shown in Fig. 6E. After 14–3-3ζ knockdown using sh140, IC_50_ value of Pro in C4-2B cells got increased, suggesting a decreased cytotoxicity of Pro after 14–3-3ζ knockdown (Fig. 6F) Similarly, after 14–3-3ζ knockdown, Luc intensity was not significant downregulated by Pro in MC3T3-ORE co-cultured with C4-2B-sh140 cells (Fig. 6G). Furthermore, the growth of PCa cells in mice tibias slowed down and the therapeutic effect of Pro was diminished after 14–3-3ζ knockdown (Fig. 6H).

### Blockade of TGF-β1/C-Raf/ MAPK feedback loop by Procoxacin/14–3-3ζ binding

**Fig. 7:**
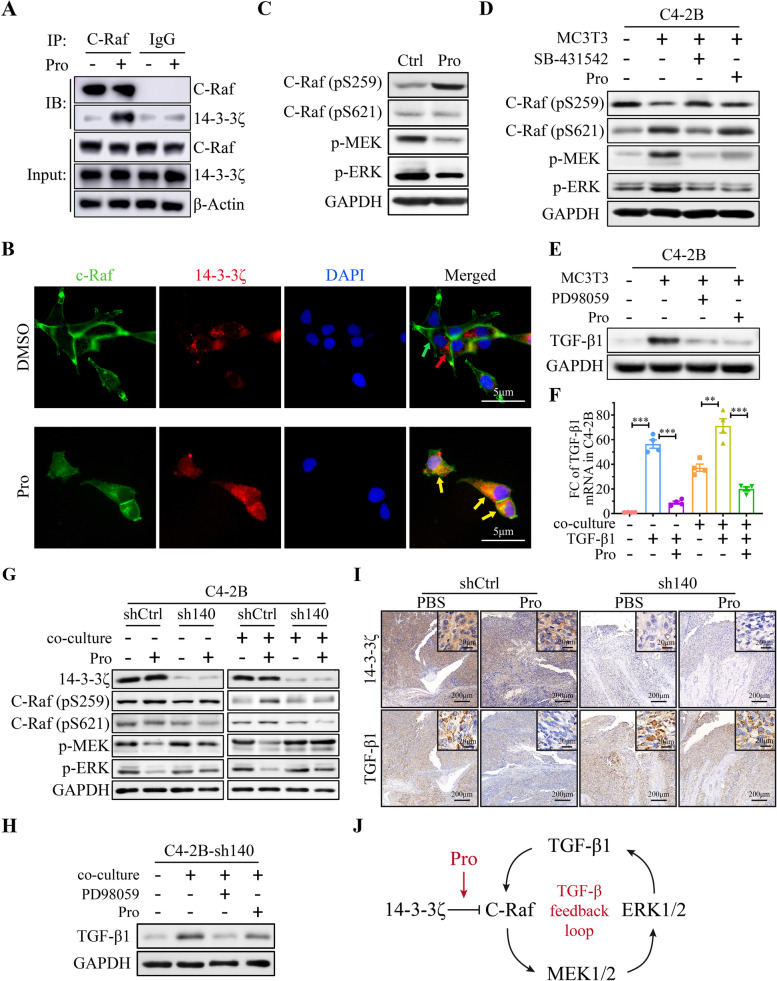
Pro enhances the inhibitory binding of 14–3-3ζ to C-Raf and blocks the positive feedback loop of TGF-β1 in OB-educated PCa. **A** Immunoprecipitation of 14–3-3ζ by C-Raf in C4-2B cells treated with DMSO or Pro (400 nM). IgG was used as the negative control. **B** Representative IF images of subcellular location of 14–3-3ζ and C-Raf in C4-2B cells treated with DMSO or Pro (400 nM). Green and red arrows indicate the separate locations of 14–3-3ζ and C-Raf; yellow arrows indicate the co-localization of 14–3-3ζ and C-Raf. **C** Western blot of the phosphorylation level of proteins in C-Raf/MAPK pathway in C4-2B cells treated with DMSO or Pro (400 nM). **D** Western blot of the phosphorylation level of indicated proteins in co-cultured C4-2B cells treated with DMSO, SB-431542 (10 μM) or Pro (400 nM). **E** Western blot of TGF-β1 in co-cultured C4-2B cells treated with DMSO, PD98059 (10 μM) or Pro (400 nM). **F** Fold change of TGF-β1 mRNA in C4-2B cells cultured alone and co-cultured with MC3T3 and treated with TGF-β1 (10 ng/ml) and Pro (400 nM). **G** Western blot of the phosphorylation level of proteins in C-Raf/MAPK pathway with or without 14–3-3ζ knockdown under indicated conditions. **H** Western blot of TGF-β1 in C4-2B-sh140 cell treated with DMSO, PD98059 (10 μM) or Pro (400 nM). **I** IHC staining of 14–3-3ζ and TGF-β1 in serial bone sections. **J** Schematic of Pro binding to 14–3-3ζ and blocking the positive feedback loop of TGF-β. ***P < 0.001

14–3-3ζ is one of the seven members of 14–3-3 adapter proteins, which regulate a diversity of cellular functions by mediating protein–protein interactions [32, 33]. We used co-immunoprecipitation to examine the interaction between 14–3-3ζ and C-Raf in C4-2B cell lysate and found that significantly more 14–3-3ζ was pulled down by C-Raf in Pro-treated samples than in DMSO-treated samples (Fig. 7A). Furthermore, immunofluorescence (IF) images showed the co-localization of 14–3-3ζ and C-Raf in C4-2B cells after Pro treatment (Fig. 7B). These results demonstrate that insertion of Pro into Raf/14–3-3ζ complex promotes the binding of 14–3-3ζ to C-Raf.

A previous study has reported that the phosphorylation-dependent binding of 14–3-3ζ to C-RAF's Ser233 and Ser259 prevents Ras-mediated activation of C-Raf/ERK MAPK pathway, whereas binding of 14–3-3ζ to Ser621 plays an activating role [34, 35]. Under directly cultured conditions, Pro significantly increased the phosphorylation of C-Raf on Ser259 but not Ser621 in C4-2B cells and downregulated the phosphorylated MEK1/2 and ERK1/2, indicating the suppressive effect of Pro on C-Raf/ERK MAPK pathway (Fig. 7C). When co-cultured with MC3T3-E1, pS259/pS621 ratio of C-Raf was decreased and ERK MAPK pathway was activated in C4-2B cells. Blockade of TGF-β signaling by TGF-β type I receptor (TβRI) inhibitor, SB-431542, reversed the pS259/pS621 ratio which inactivated C-Raf/ERK MAPK pathway. This result indicates that the OB-induced activation of C-Raf/ERK MAPK pathway is TGF-β-dependent. In contrast, Pro did not reverse the pS259/pS621 ratio in co-cultured C4-2B cells but still inactivated the downstream ERK MAPK pathway (Fig. 7D).

On the other hand, we tested if the excessive amount of TGF-β1 was a result of the overactivation of C-Raf/ERK MAPK pathway. At the protein level, inhibition of MEK1/2 and ERK1/2 by PD98059 effectively suppressed the elevation of TGF-β1 in co-cultured C4-2B cells, resembling the effect of Pro (Fig. 7E). At the mRNA level, we found that addition of exogenous TGF-β1 in the cell cultures could induce the transcription of TGF-β1 itself, and this could be suppressed by Pro in either direct culture or co-culture conditions (Fig. 7F). There results suggest that a positive feedback loop exists in the regulation of TGF-β in the PCa-OB microenvironment.

Next, we confirmed the role of 14–3-3ζ in regulating the TGF-β/C-Raf/MAPK positive feedback loop. Knockdown of 14–3-3ζ counteracted the suppressive effect of Pro on C-Raf/ERK MAPK pathway in C4-2B cells and this effect is more evident under co-cultured condition (Fig. 7G). In addition, after 14–3-3ζ knockdown, PD98059 could still inhibit OB-induced TGF-β1 transcription in C4-2B cells, while the effect of Pro was mitigated (Fig. 7H). Consistently, IHC staining on serial bone sections revealed that TGF-β1 expressed by bone-metastatic PCa cells was no longer significantly suppressed by Pro after 14–3-3ζ knockdown (Fig. 7I). All together, these results indicate that Pro blocks the positive feedback loop of TGF-β1 in PCa-OB microenvironment by promoting the binding of 14–3-3ζ to inhibitory phosphorylation site of C-Raf (Fig. 7J).

### Prevention of metastasis of both osteogenic and osteolytic PCa by Procoxacin

**Fig. 8:**
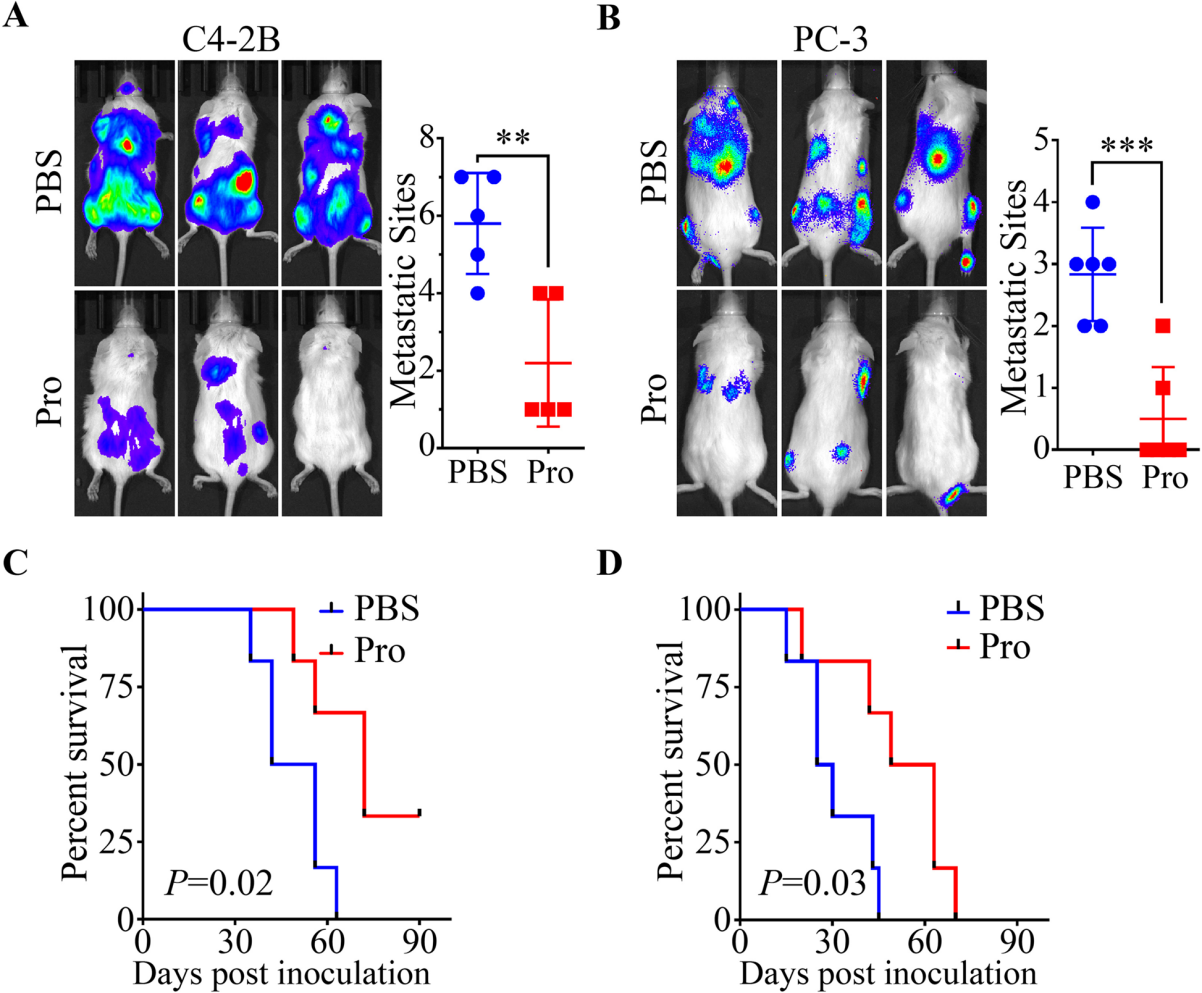
Pro pretreatment prevents metastasis of PCa cells in vivo. **A** and **B** Representative images of mice pretreated with PBS or Pro (1 mg/kg/day for 3 days) followed by C4-2B (n=5) (**A**) or PC-3 (n=6) (**B**) injection through left ventricle. Pictures were taken 3 weeks after inoculation. Number of metastases was counted on the right. **C** and **D** Mice used in the previous step were treated with PBS or Pro (2 mg/kg). Survival rate of mice bearing C4-2B (**C**) and PC-3 (**D**) was calculated. **P < 0.01, ***P < 0.001

At last, we investigated the effect of Pro on mPCa metastasis using a mouse intracardiac injection model. As shown in Fig. 8A and B, Pro significantly reduced the number of metastases formed by both the osteogenic C4-2B cells and osteolytic PC-3 cells. Survival analysis (Fig. 8C and D) revealed that Pro effectively prolonged the survival time of NOD-SCID mice received intracardiac cancer injection.

## Discussion

Cancer-host interaction is considered an important promoter of cancer progression and has been recognized as a therapeutic target. In this study, we established a drug screening system based on C4-2B-MC3T3 cell co-culture and evaluated the effects of 631 compounds on PCa-OB interaction. OSE2 is a specific binding site of the transcriptional factor RUNX2 [36] and ALP is an enzyme expressed on the surface of osteoblasts and has been clinically used as a biomarker for bone-metastatic PCa [37, 38]. We find that the combination of OSE2 copies and *Alp* promoter in the reporter gene can sensitively reflect the activation of OBs. In addition, during drug screening, we also evaluated the toxicity of the compounds to C4-2B and MC3T3 cells, to select drugs that can inhibit PCa cells without damaging normal stem cells in bone. In this way, toxic compounds can be excluded and the side effects of selected compounds can be minimized.

Although the overactivation of OBs plays a dominant role in bone metastasis of PCa, OCs may also be essential in disease progression. Firstly, OC-mediated bone resorption provides the prerequisite for cancer cells to home to the rigid bone compartments. Moreover, during bone formation, a portion of cytokines (such as TGF-β) produced by osteoblastic lineage cells is embedded in the bone matrix and at the initial stage of PCa entering the bone, both OB and OCs are activated. This not only leads to the increase of cytokine secretion by OBs, but also promotes the release of cytokines embedded in the bone matrix through OC-mediated osteolysis [2, 39]. In this study, Pro simultaneously exerts drug effects on both OBs and OCs, and rebuilds the balance between osteogenesis and osteolysis at concentrations around 100 nM, which indicates that Pro may be of great potential for clinical application. It is noteworthy that, anti-osteoclasts agents, such as Denosumab and Zoledronic Acid, are the first-line in current treatment for late-stage mPCa, though they have no effect on OBs and cannot improve survival rates of patients.

TGF-β is one of the most important factors in the bone microenvironment and has been regarded as a key regulator in bone overturn and bone metastasis [40–42]. Autocrine and paracrine stimulation by TGF-β is important in the maintenance and differentiation of OB progenitors [39]. The aberrant elevation of TGF-β in the bone microenvironment is associated with the disruption of the osteogenic-osteolytic balance and cancer progression [43]. Our results show that Pro effectively blocks the positive feedback loop of TGF-β in PCa-OB microenvironment and the inhibitory effect of Pro on OC may further decrease the TGF-β level in the bone metastasis of PCa.

In addition, we first explored the molecular target of Pro and identified 14–3-3ζ as one of the drug targets. In mPCa, the frequency of 14–3-3ζ amplification has been reported to be higher compared with primary PCa [44], which may be associated with poor prognosis [45]. Additionally, a small molecule inhibitor targeting 14–3-3ζ was demonstrated to be effective in treating PCa [46]. We find that the binding of Pro to 14–3-3ζ prevents the disassociation of the 14–3-3ζ/C-Raf complex and maintains the inhibitory phosphorylation (Ser259) of C-Raf, which further inactivates the TGF-β/C-Raf/ERK MAPK pathway and inhibits TGF-β production.

However, some limitations also exist in this study. Additional mechanisms by which Pro exerts its effect on OBs and OCs may exist. Further experiments are needed to explore the mechanisms of Pro in the cytotoxicity towards OCs. Moreover, given that natural compounds may have multiple targets leading to various biological activities [47, 48], there may be other drug targets of Pro besides 14–3-3ζ, such as FKBP/mTOR complex, protein phosphatase PP1, et al. It is also plausible that some of the biological activities of Pro may be achieved through its role as a cation transporter [49]. However, this is out of the scope of the present study.

## Conclusion

This study demonstrated the reliability and feasibility of a drug screening strategy based on cancer-host interaction and showed that Pro might be a potential therapeutic agent in the treatment of bone-metastatic PCa by blocking the TGF-β feedback loop in the bone metastasis.

## Supplementary Information

**Additional file 1:** **Fig. S1.** Changes in candidate reporter genes and compounds validation. (A, B and C) Fold change of mRNA level of Runx2 (A), Opn (B) and Col1a1 (C) in directly cultured or co-cultured MC3T3-E1 cells in PM at different time points compared with Day 0. (D, E, F and G) Cell viability of pre-osteoblast cell lines MC3T3-E1 (D) and C3H10T1/2 (E) and bone-derived PCa cell lines C4-2B (F) and PC-3 (G) treated with indicated compounds (0.5 μM or 2.5 μM). 

**Additional file 2:** **Fig. S2.** Effet of Pro on OBs and PCa cells. (A) Compounds that suppress the viability of C4-2B cells have different effects on RLI. (B) Viability of C4-2B cells treated with Pro at indicated concentrations.

**Additional file 3:** **Fig. S3.** Quantification of Alizarin Red S staining in Figure 2D (A) and 2E (B). **P <0.01.

**Additional file 4:** **Fig. S4.** Representative images of CK8/18 and H&E staining of PCa-involved tibias. (A) Immunohistochemical staining of CK8/18 in PCa-involved tibias. (B) Representative image of H&E staining in a C4-2B-inoculated tibia with large fluorescence range.

**Additional file 5:** **Fig. S5.** Effect of Pro on the expression of TGF-β2 in MC3T3-E1 cells. Western blot of TGF-β2 in MC3T3-E1 cells co-cultured with C4-2B in 0.4 μm transwells under indicated conditions.

**Additional file 6:** **Fig. S6.** Predicted structure of Pro binding to FKBP/mTOR complex. The carboxylic end of Pro was anchored on FKBP5 by inserting into a hydrophobic cavity, mainly delineated by aromatic side chains.

**Additional file 7: Table S1. Primers for real-time PCR.**

**Additional file 8: Table S2. Data of the compound screening.**

## Data Availability

All data underlying the study are available from the corresponding authors on reasonable request.

## Abbreviations

PCa: Prostate cancer
mPCa: Metastatic prostate cancer
OB(s): Osteoblast(s)
OC(s): Osteoclast(s)
OSE2: osteoblast-specific cis-acting element
Pro: Procoxacin
PM: Promineralization medium
ORE: Osteogenesis-Response Element
GFP: Green fluorescence protein
RFP: Red fluorescence protein
Luc: Luciferin luminescence
RGI: Relative green fluorescence intensity
RRI: Relative red fluorescence intensity
RLI: Relative luciferin luminescence intensity
AIC: AICAR (phosphate)
Gin: Ginsenoside Rg2
Ber: bergapten
Des: Desaminotyrosine
Rot: Rotundine
Kea: Kaempferol
PFA: paraformaldehyde
GEO: Gene Expression Omnibus
WB: Western Blot
IHC: Immunohistochemistry
IF: Immunofluorescence
PBS: Phosphate-buffered saline
DMSO: Dimethyl sulfoxide
BMSCs: Bone marrow-derived mesenchymal stem cells
BMM: Bone marrow macrophages
M-CSF: Macrophage colony stimulating factor
PDB: Protein Data Bank
SPR: Surface plasmon resonance
RU: Response unit
