# Supplementary figures and images for: Procoxacin bidirectionally inhibits osteoblastic and osteoclastic activity in bone and suppresses bone metastasis of prostate cancer
Source: J Exp Clin Cancer Res. 2023 Feb 9;42:45. doi: 10.1186/s13046-023-02610-7 (PMC9909988; doi:10.1186/s13046-023-02610-7)

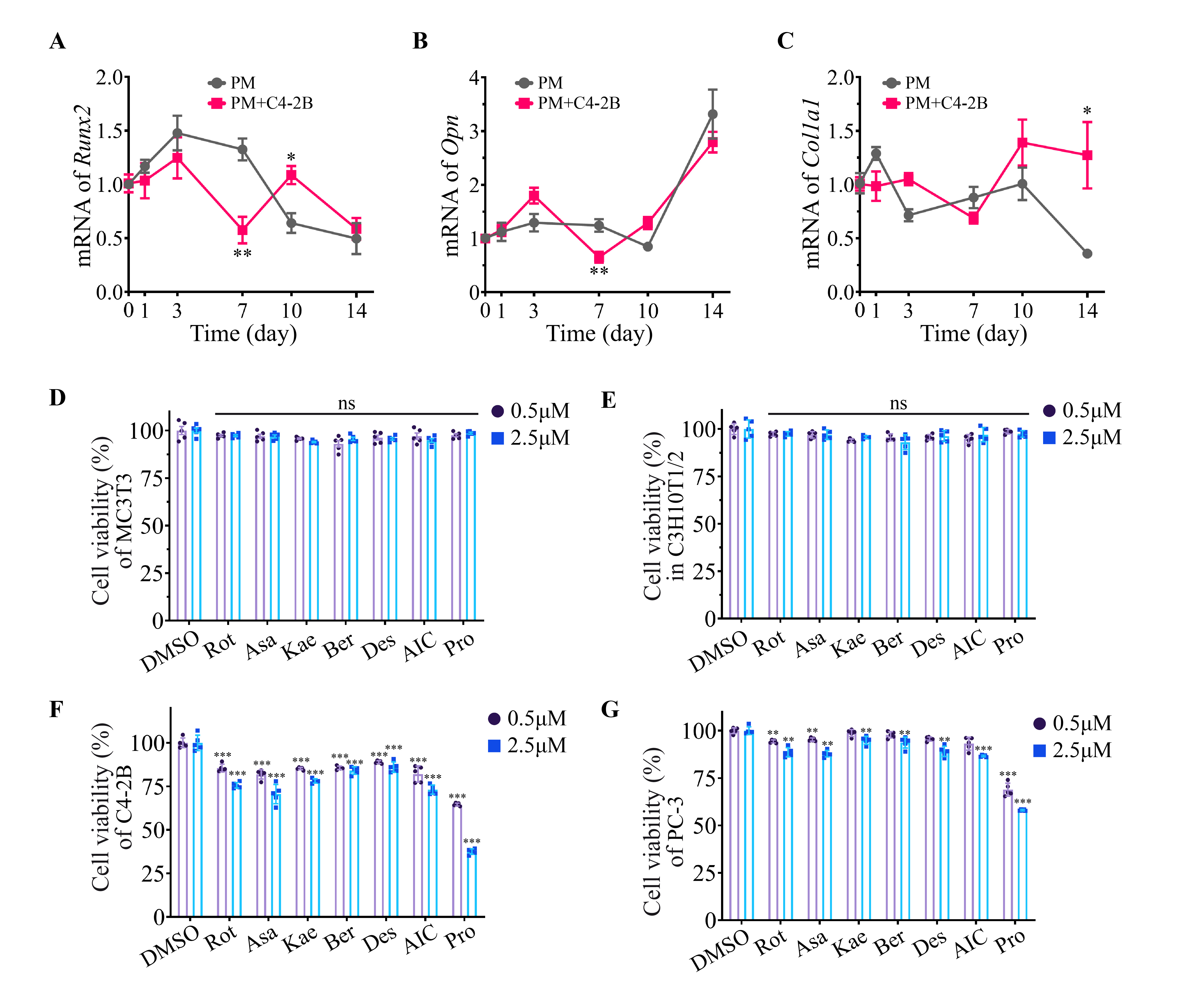

Supplement: Supplementary file 1 — Additional file 1: Fig. S1. Changes in candidate reporter genes and compounds validation. (A, B and C) Fold change of mRNA level of Runx2 (A), Opn (B) and Col1a1 (C) in directly cultured or co-cultured MC3T3-E1 cells in PM at different time points compared with Day 0. (D, E, F and G) Cell viability of pre-osteoblast cell lines MC3T3-E1 (D) and C3H10T1/2 (E) and bone-derived PCa cell lines C4-2B (F) and PC-3 (G) treated with indicated compounds (0.5 μM or 2.5 μM). [file 13046_2023_2610_MOESM1_ESM.jpg]

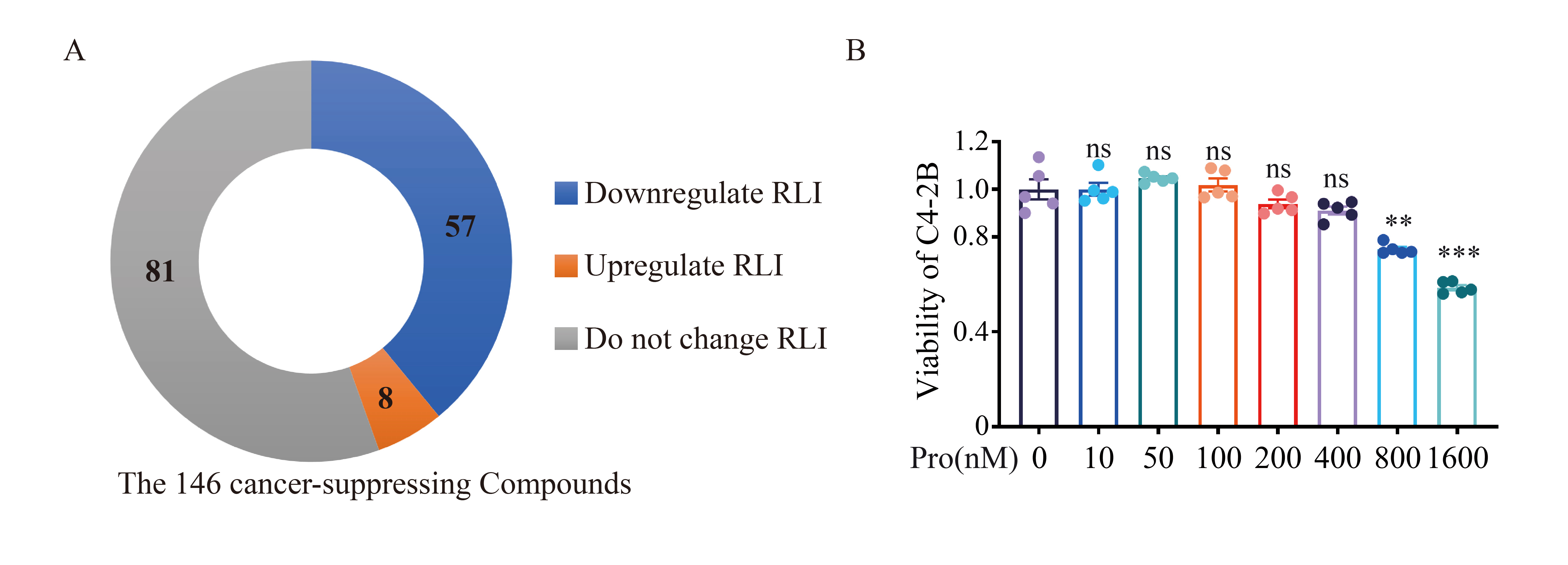

Supplement: Supplementary file 2 — Additional file 2: Fig. S2. Effet of Pro on OBs and PCa cells. (A) Compounds that suppress the viability of C4-2B cells have different effects on RLI. (B) Viability of C4-2B cells treated with Pro at indicated concentrations. [file 13046_2023_2610_MOESM2_ESM.jpg]

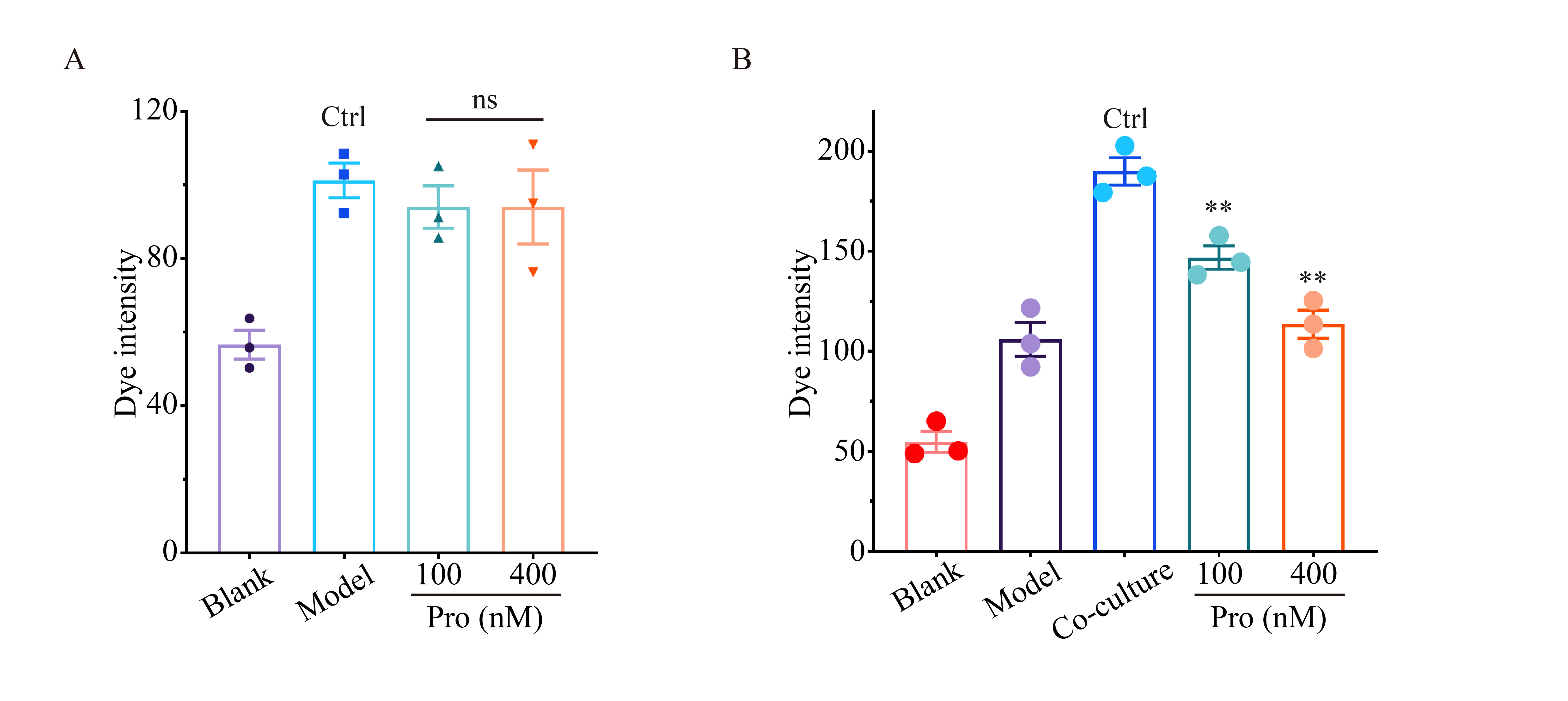

Supplement: Supplementary file 3 — Additional file 3: Fig. S3. Quantification of Alizarin Red S staining in Figure 2D (A) and 2E (B). **P <0.01. [file 13046_2023_2610_MOESM3_ESM.jpg]

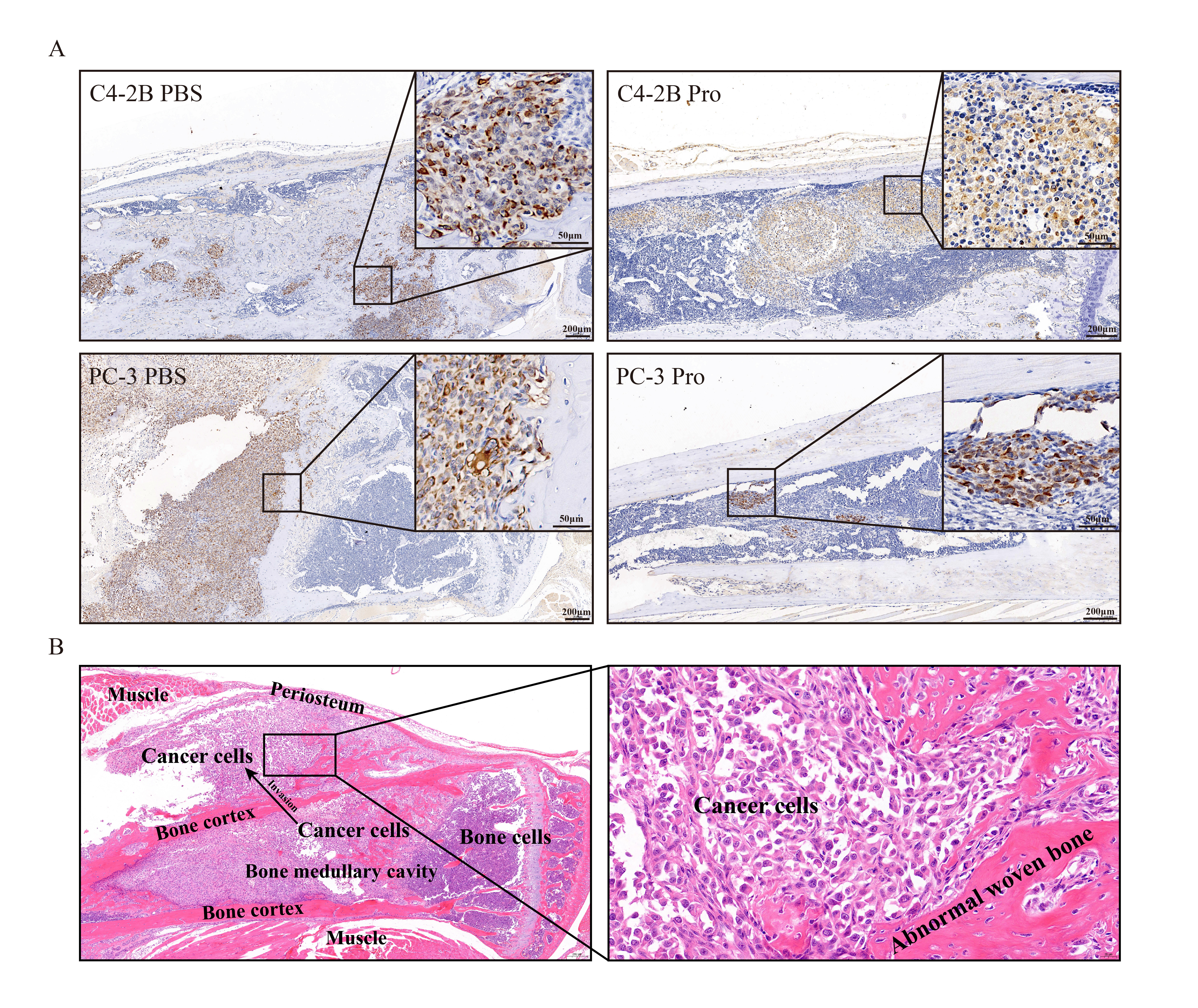

Supplement: Supplementary file 4 — Additional file 4: Fig. S4. Representative images of CK8/18 and H&E staining of PCa-involved tibias. (A) Immunohistochemical staining of CK8/18 in PCa-involved tibias. (B) Representative image of H&E staining in a C4-2B-inoculated tibia with large fluorescence range. [file 13046_2023_2610_MOESM4_ESM.jpg]

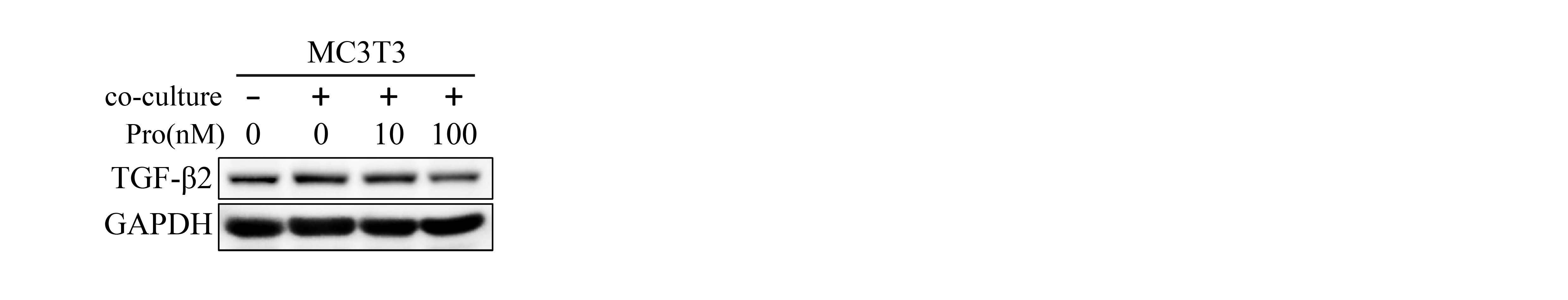

Supplement: Supplementary file 5 — Additional file 5: Fig. S5. Effect of Pro on the expression of TGF-β2 in MC3T3-E1 cells. Western blot of TGF-β2 in MC3T3-E1 cells co-cultured with C4-2B in 0.4 μm transwells under indicated conditions. [file 13046_2023_2610_MOESM5_ESM.jpg]

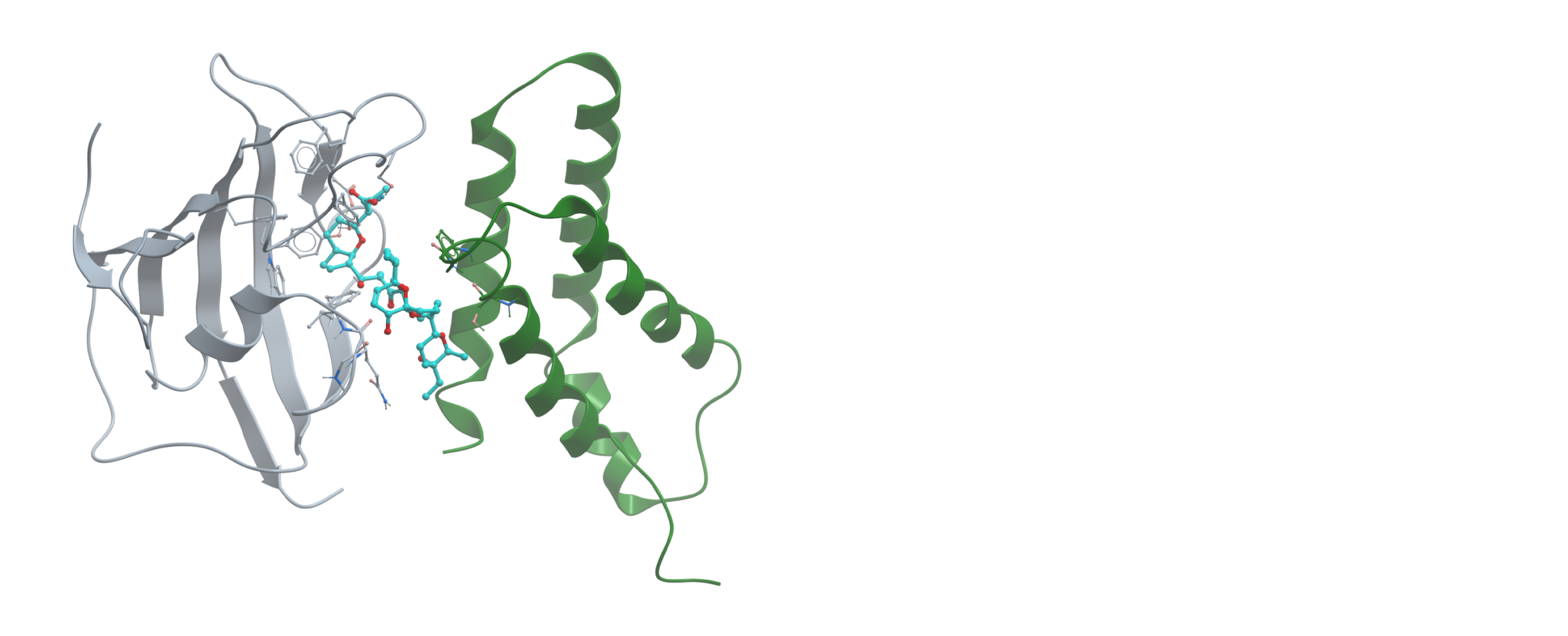

Supplement: Supplementary file 6 — Additional file 6: Fig. S6. Predicted structure of Pro binding to FKBP/mTOR complex. The carboxylic end of Pro was anchored on FKBP5 by inserting into a hydrophobic cavity, mainly delineated by aromatic side chains. [file 13046_2023_2610_MOESM6_ESM.jpg]
